# Synthesis of Homo‐Metallic Heavier Analogues of Cyclobutene and the Cyclobutadiene Dianion**

**DOI:** 10.1002/chem.202300006

**Published:** 2023-02-27

**Authors:** Xiongfei Zheng, Agamemnon E. Crumpton, Andrey V. Protchenko, Mathias A. Ellwanger, Andreas Heilmann, Simon Aldridge

**Affiliations:** ^1^ Inorganic Chemistry Laboratory Department of Chemistry University of Oxford South Parks Road OX1 3QR Oxford UK

**Keywords:** aromaticity, cyclobutadiene dianion, quantum chemical calculations, sub-valent compounds, tin

## Abstract

The reduction of the boryl‐substituted Sn^II^ bromide {(HCDippN)_2_B}Sn(IPrMe)Br with 1.5 equivalents of potassium graphite leads to the generation of the cyclic tetratin tetraboryl system K_2_[Sn_4_{B(NDippCH)_2_}_4_], a homo‐metallic heavier analogue of the cyclobutadiene dianion. This system is non‐aromatic as determined by Nucleus Independent Chemical Shift Calculations (NICS(0)=−0.28, NICS(1)=−3.17), with the primary contributing resonance structures shown by Natural Resonance Theory (NRT) to involve a Sn=Sn double bond and 1,2‐localized negative charges. Abstraction of the K^+^ cations or oxidation leads to contraction or cleavage of the Sn_4_ unit, respectively, while protonation generates the neutral dihydride 1,2‐Sn_4_{B(NDippCH)_2_}_4_H_2_ (a heavier homologue of cyclobutene) in a manner consistent with the predicted charge distribution in the [Sn_4_{B(NDippCH)_2_}_4_]^2−^ dianion.

## Graphical abstract

**The reduction of** {(HCDippN)_2_B}Sn(IPrMe)Br with 1.5 equivalents of KC_8_ generates the cyclic tetra‐tin tetra‐boryl system K_2_[Sn_4_{B(NDippCH)_2_}_4_], a homo‐metallic heavier analogue of the cyclobutadiene dianion. This system is non‐aromatic as determined by Nucleus Independent Chemical Shift Calculations, with the principal resonance structures shown to involve a Sn=Sn double bond and 1,2‐localized negative charges.

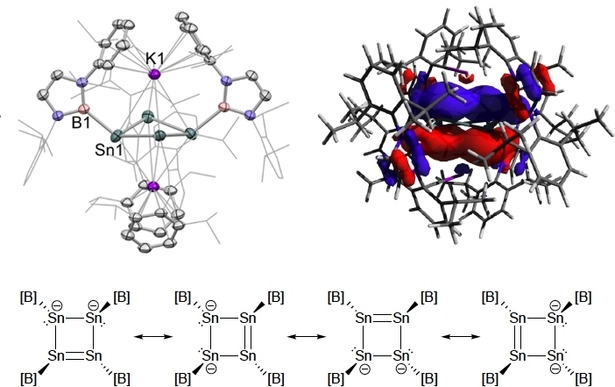

## Introduction

**Figure 1:**
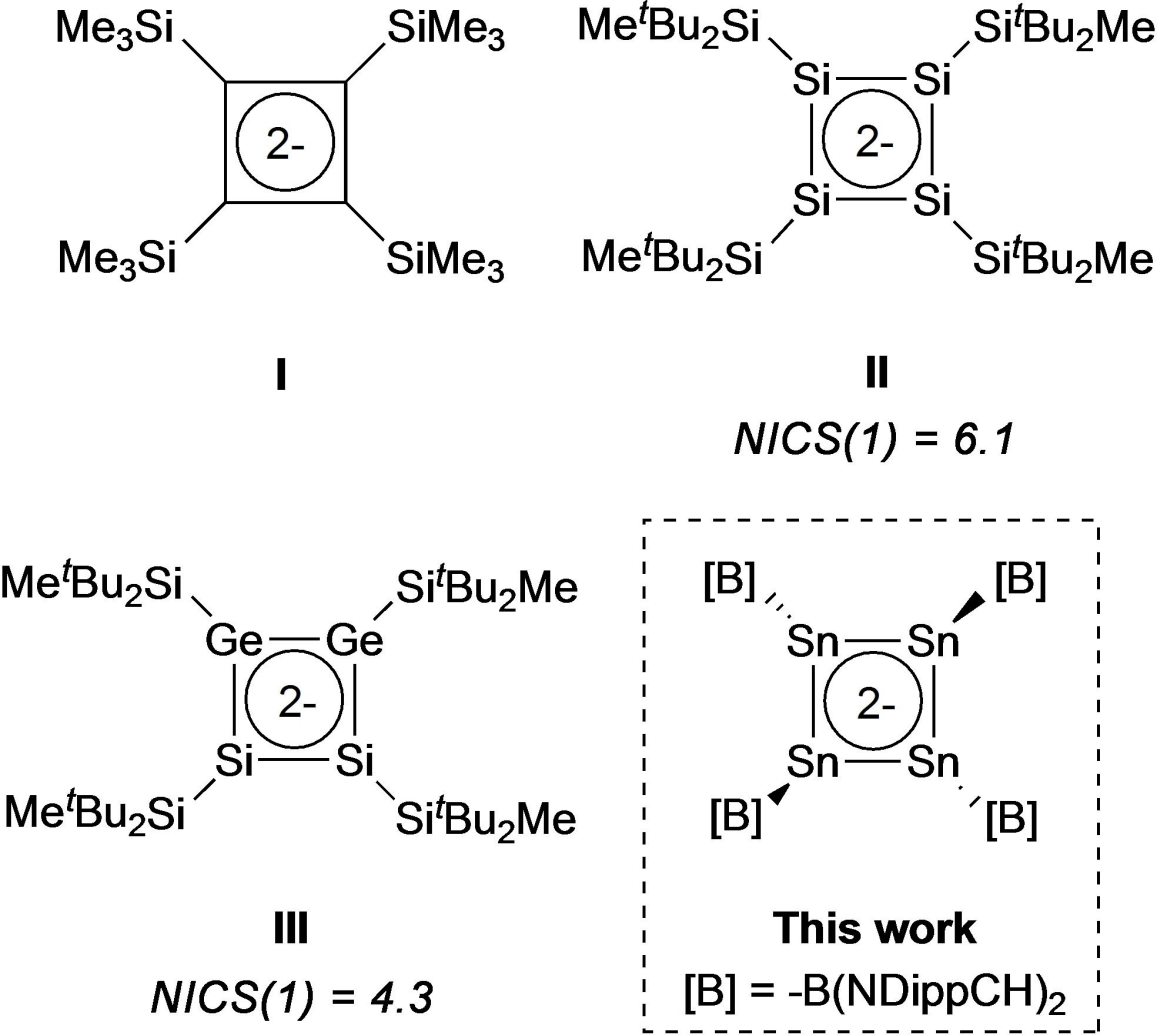
Dianionic derivatives of cyclobutadiene and their heavier analogues of relevance to the current study.

The concept of aromaticity is a key tool in understanding structure and reactivity in organic chemistry.^[1]^ Extrapolation to the heavier elements of group 14 brings with it additional challenges relating not only to synthetic strategy, but also to the rationalization of geometric data in terms of electronic structure.^[2]^ At a superficial level, Huckel's rule predicts that both the dianion and dication of cyclobutadiene (CBD) should be aromatic with 6π and 2π electrons, respectively. However, the potential aromaticity of these systems has attracted significant debate; doubly anionic 6π cyclic systems have been predicted to behave quite differently from neutral and singly charged analogues because of additional Coulombic repulsion.[^3^, ^4^] A folded [CBD]^2−^ structure with one localized and one allylic delocalized negative charge,^[5]^ a trapezoid structure with 1,2‐localized negative charges and a C=C double bond,^[6]^ and a planar delocalized structure stabilized by the coordination of two Li cations,[^7^, ^8^] have all been evaluated via quantum chemical methods. Experimentally, there have been studies on the transient cyclobutadiene dianion^[7]^ and its derivatives stabilized by ester or phenyl groups.[^10^, ^11^] The first cyclic system featuring a delocalized 6π‐electron system was characterized crystallo‐graphically by Sekiguchi et al. in 2000 (**I**, Figure 1).^[12]^ Subsequently in 2004, the same group isolated [CBD]^2−^ derivatives of the heavier group 14 elements ([R_4_Si_4_]^2−^, **II**, and [R_4_Si_2_Ge_2_]^2−^, **III**; R=SiMe^*t*^Bu_2_)^[13]^ in which the central four‐membered ring is significantly folded, and features two η^2^‐1,3‐coordinated potassium cations. These systems are thought to be non‐aromatic on the basis of nucleus‐independent chemical shift (NICS) calculations.

We have recently been interested in the use of strongly σ‐donating and sterically encumbered boryl ligands, −B(NDippCH)_2_ for the stabilization of main group systems featuring unusual electronic or geometric structure, and unprecedented modes of reactivity.^[14]^ These include a germanium analogue of vinylidene {(HCDippN)_2_B}_2_Ge=Ge,^[14h]^ and a 2π‐electron tetra‐indium dianion [In_4_{B(NDippCH)_2_}_4_]^2−^ (**IV**, as the dipotassium salt) which shows a moderate degree of aromatic character.^[14j]^ Here we show that a superficially similar tin compound K_2_[Sn_4_{B(NDippCH)_2_}_4_] can be accessed via controlled (stoichiometric) reduction of a boryltin(II) precursor. This system represents the first tin‐containing analogue of [CBD]^2−^, but in contrast to its indium counterpart, is non‐aromatic in nature.

## Results and Discussion

**Scheme 1:**
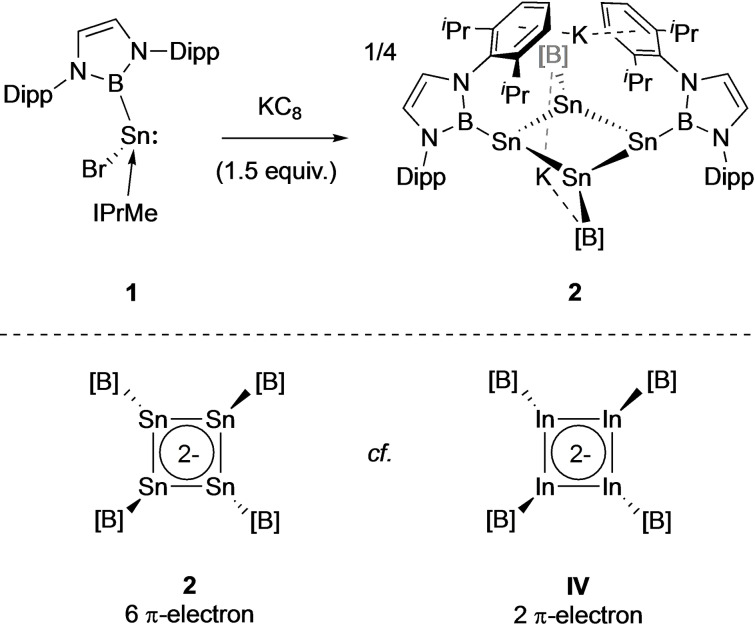
Reduction of NHC‐supported boryl‐tin bromide **1** with 1.5 equiv. of potassium graphite to generate cyclic tetratin tetraboryl system K_2_[Sn_4_{B(NDippCH)_2_}_4_] (**2**). ([B]={B(NDippCH)_2_}; Dipp=C_6_H_3_^*i*^Pr_2_‐2,6).

**Figure 2:**
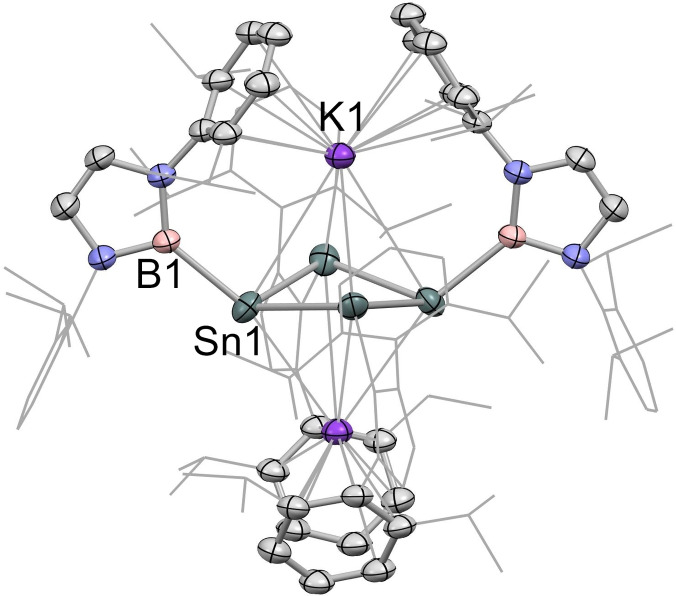
Molecular structure of K_2_[Sn_4_{B(NDippCH)_2_}_4_] (**2**) in the solid state as determined by X‐ray crystallography. Hydrogen atoms omitted and selected groups shown in wireframe format for clarity; thermal ellipsoids drawn at the 40 % probability level. Key bond lengths (Å) and angles (°): Sn−Sn 2.9018(9), 2.9010(6); Sn−B 2.306(5); Sn⋅⋅⋅K 3.915, 3.510; Sn−Sn−Sn 88.41(3); centroid‐Sn−B 132.8.

The reduction of {(HCDippN)_2_B}Sn(IPrMe)Br (**1**; Dipp=2,6‐^*i*^Pr_2_C_6_H_3_; IprMe=C{(N^*i*^PrCMe)_2_})^[15]^ with 1.5 equiv. of potassium graphite leads to the formation of a diamagnetic product characterized by a single ^11^B NMR resonance at δ_B_=49 ppm. The ^1^H NMR spectrum suggests a lower symmetry for the ligand scaffold, showing four ^*i*^Pr CH_3_ doublets and two CH septets, in addition to two mutually coupled doublets for the CH groups of the boryl heterocycle. Black crystals grown from toluene/pentane solution allow definitive characterization of the product to be achieved by X‐ray crystallography, and shows that it is the cyclic tetratin tetraboryl system K_2_[Sn_4_{B(NDippCH)_2_}_4_] (**2**) (Scheme 1 and Figure 2).

The solid‐state structure of **2** features an approximately square array of four (symmetry‐related) tin atoms, with each metal centre being additionally bound to a single terminal boryl ligand (*d*(Sn−Sn)=2.9018(9), 2.9010(6) Å, *d*(Sn−B)=2.306(5) Å), and the internal Sn−Sn−Sn angles being close to 90° (88.41(3)°). The structure is completed by two K^+^ counter‐ions positioned above and below the Sn_4_ unit (K⋅⋅⋅Sn distances: 3.915 and 3.510 Å), which are sandwiched between the flanking Dipp aryl rings of diagonally opposite boryl ligands (with K⋅⋅⋅arene contacts in the range 3.088–3.413 Å). The Sn_4_ unit is puckered, with each tin atom lying 0.24 Å out of the least‐squares plane, such that the Sn_4_ centroid‐Sn−B angles are non‐linear (132.8°), and alternate boron atoms are positioned above/below the approximate Sn_4_ plane. As a consequence each tin centre is pyramidalized, with the sum of the Sn−Sn−Sn and Sn−Sn−B angles being 318.6°. By means of comparison, the corresponding angles in tetra‐silicon system **II** reported by Sekiguchi and co‐workers sum to 341/326° (for the two distinct silicon centres).^[13]^

In a broader sense, **2** represents the formal dimer of the radical anion [Sn_2_{B(NDippCH)_2_}_2_]⋅^−^, the terphenyl analogue of which, [Sn_2_Ar^Dipp^_2_]⋅^−^, has been reported by Power and co‐workers (Ar^Dipp^=C_6_H_3_Dipp_2_‐2,6).^[16]^ From a synthetic perspective, it is noteworthy that **2** can also be formed by one‐electron oxidation of [Sn_2_{B(NDippCH)_2_}_2_]^2−[15]^ using trityl oxidants. From a structural viewpoint, the underlying difference between tetranuclear **2**, and the dimeric terphenyl systems presumably relates to the smaller steric profile of the boryl ligand. The longer Sn−B bond (cf. Sn−C) and five‐ (rather than six‐) membered central heterocycle cause the pendant Dipp groups to exert a smaller steric profile at the tin centre.

Geometrically, the structure of **2** bears a close resemblance to the corresponding indium compound K_2_[In_4_{B(NDippCH)_2_}_4_] (**IV**, Scheme 1), which possesses four electrons fewer within a less puckered [In_4_{B(NDippCH)_2_}_4_]^2−^ unit.^[14j]^ In the indium system, the four (symmetry‐equivalent) metal centres are less significantly displaced from the least‐squares plane (0.12 Å), and Nucleus Independent Chemical Shift calculations are consistent with moderately aromatic character attributable to the two π‐electrons within the cyclic manifold. The corresponding unit in **2** could also give rise to Hückel aromaticity on the basis of the six π‐electron count, and we therefore set out to examine the electronic structure of **2** by quantum chemical methods.

**Figure 3:**
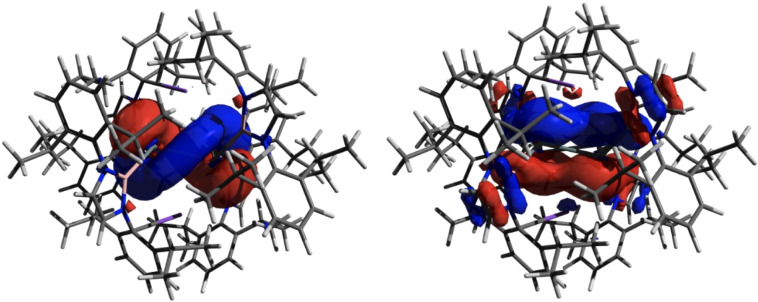
Electron density surfaces of key molecular orbitals for K_2_[Sn_4_{B(NDippCH)_2_}_4_] (**2**) calculated by DFT (r^2^‐scan def2‐TZVP level): (left) HOMO; (right) HOMO‐5.

CASSCF calculations (see Supporting Information) are consistent with the singlet ground state implied experimentally for **2** (with a singlet‐triplet gap of 0.50 eV); the associated HOMO‐LUMO separation is 1.29 eV. DFT calculations (r^2^‐scan def2‐TZVP level) reveal that π‐symmetry orbitals in **2** are primarily defined by the in‐phase HOMO‐5 (analogous to Π_1_ for [CBD]^2−^) and by a near‐degenerate pair (HOMO and HOMO‐1) of essentially equivalent form reminiscent of Π_2_/Π_3_ for [CBD]^2−^, but twisted due to the non‐planar nature of the Sn_4_ unit (Figure 3. The highest energy molecular orbital possessing Sn−Sn σ‐bonding character is the HOMO‐2. Nucleus independent chemical shift (NICS) values have been calculated for **2** (NICS(0)=−0.28, NICS(1)=−3.17) and can be compared with the values calculated using the same method for ′moderately aromatic′ K_2_[In_4_{B(NDippCH)_2_}_4_] (**IV**; NICS(0)=−7.52, NICS(1)=−8.61), implying that the degree of aromatic character in **2** is minimal. This finding is consistent with the non‐aromatic nature of the 6π‐electron silicon and germanium systems **II** and **III**, and presumably reflects the markedly pyramidal nature of the tin centres in **2**. The contrasting signs of the curvature of the *z*‐component of the electron density at the ring critical points (RCPs) in tetra‐indium system **IV** (−0.087 e Å^−5^) and tetra‐tin compound **2** (+0.024 e Å^−5^) also provide further evidence for their aromatic/non‐aromatic nature.

**Scheme 2:**
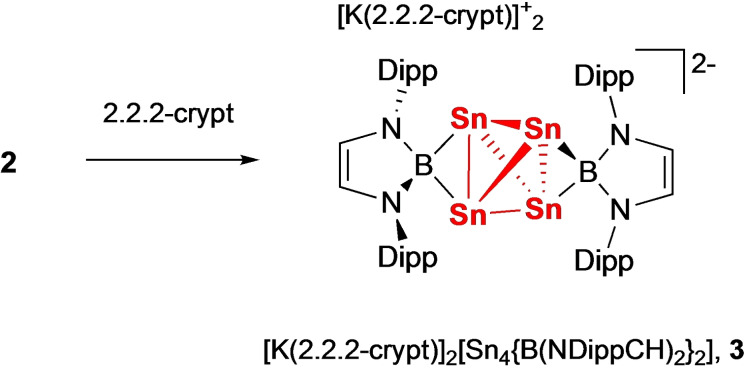
Cation abstraction from **2** by 2.2.2‐crypt leading to formation of the *nido* cluster [K(2.2.2‐crypt)]_2_[Sn_4_{B(NDippCH)_2_}_2_] (**3**).

**Figure 4:**
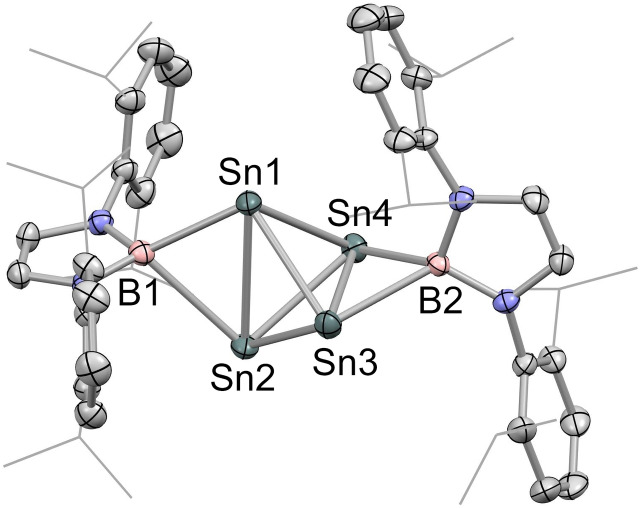
Molecular structure of the dianionic component of [K(2.2.2‐crypt)]_2_[Sn_4_{B(NDippCH)_2_}_2_] (**3**) in the solid state as determined by X‐ray crystallography. Cations and hydrogen atoms omitted and selected groups shown in wireframe format for clarity; thermal ellipsoids drawn at the 40 % probability level. Key bond lengths (Å): Sn−Sn 3.1279(6), 2.8911(7), 2.8729(7), 2.8184(6), 2.8414(6), 3.1108(8); Sn−B1 2.737(6), 2.370(6); Sn−B2 2.618(5), 2.468(5).

Given the potential structural role of the K^+^ ions in **2** (and related systems such as **II** and **III**) we wanted to probe the consequences of cation abstraction. As such, the reaction of **2** with two equiv. of 2.2.2‐cryptand in benzene‐d_6_ solution was investigated. The product (**3**) precipitates as deep purple crystals and can be shown by X‐ray crystallography to consist of a tetrahedral Sn_4_ cluster [Sn_4_{B(NDippCH)_2_}_2_]^2−^ featuring two boryl ligands bridging opposite Sn−Sn edges, together with two [K(2.2.2‐crypt)]^+^ counter‐cations (Scheme 2 and Figure 4). **3** can be viewed as a *nido* cluster based on the Wade‐Mingos cluster electron counting rules (6 PSEPs), and results from the loss of two (formally charge neutral) boryl ligands – which are observed in situ (by ^1^H NMR) as (HCDippN)_2_BD. The formation of **3** from **2** under these conditions implies that the inclusion of the K^+^ cations is essential to the structural integrity of **2**. At a broader level, this finding is consistent with computational studies of the [CBD]^2−^ system, which suggest that electron loss should be extremely facile for the gas‐phase (cation‐free) species.^[3]^

**Scheme 3:**
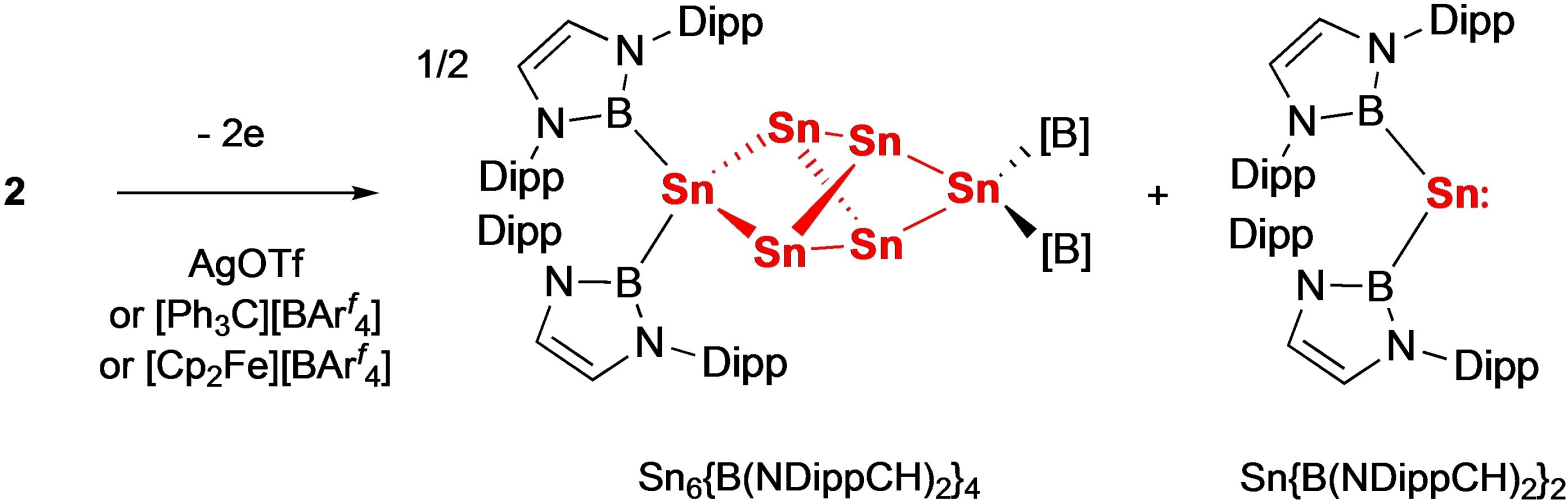
Oxidation of **2** leading to formation of a 1 : 2 mixture of Sn_6_{B(NDippCH)_2_}_4_ and Sn{B(NDippCH)_2_}_2_.^[15]^

The oxidation of **2** was also examined with the possibility of accessing related neutral (or even cationic) Sn_4_ systems. However, in line with other reports of charge neutral systems of the stoichiometry [Sn{B(NDippCH)_2_}]_*n*_ ^[15]^ (and in contrast to the related germanium systems, for which the digermavinylidene, {(HCDippN)_2_B}_2_GeGe, can be isolated),^[14h]^ disproportionation is observed in the presence of a range of oxidizing agents, leading to the formation of a 1 : 2 mixture of the cluster Sn_6_{B(NDippCH)_2_}_4_ and the bis(boryl)stannylene (Scheme 3).^[14f]^

**Scheme 4:**
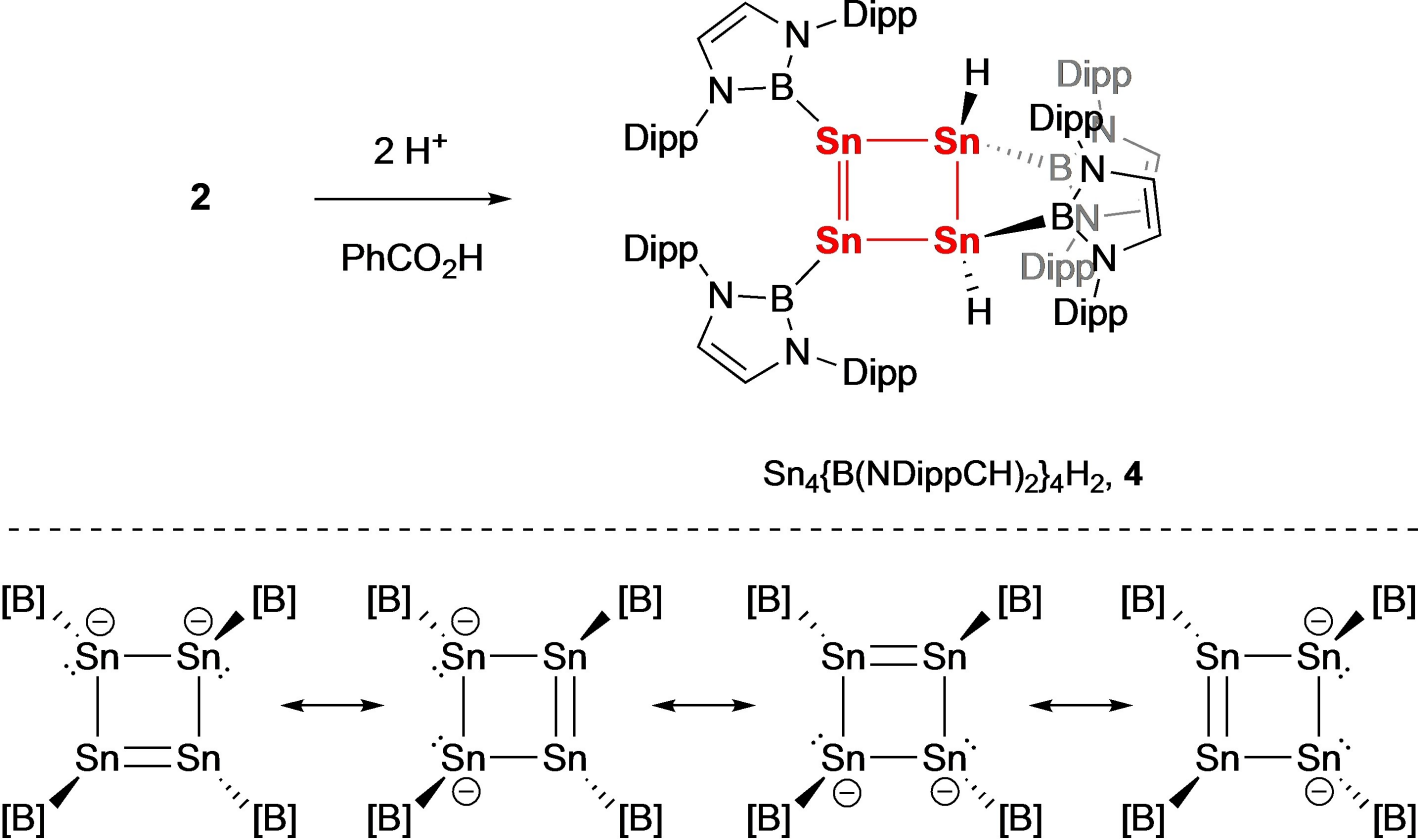
Top) Protonation of the tin core of K_2_[Sn_4_{B(NDippCH)_2_}_4_] (**2**), yielding Sn_4_{B(NDippCH)_2_}_4_H_2_ (**4**). Bottom) Major contributing resonance structures for [Sn_4_{B(NDippCH)_2_}_4_]^2−^.

**Figure 5:**
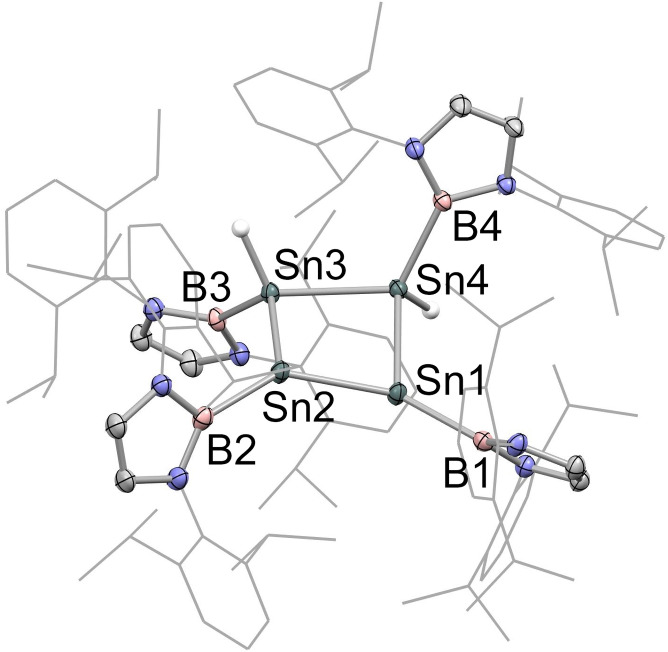
Molecular structure of Sn_4_{B(NDippCH)_2_}_4_H_2_ (**4**) in the solid state as determined by X‐ray crystallography. Most hydrogen atoms omitted and selected groups shown in wireframe format for clarity; thermal ellipsoids drawn at the 40 % probability level. Key bond lengths (Å), angles (°) and torsions (°): Sn−Sn 2.6737(7), 2.8232(5), 2.8045(7), 2.8333(5); Sn−B 2.280(4), 2.273(3), 2.269(3), 2.262(4); Sn−Sn−Sn 91.01(2), 89.12(2), 88.18(2), 86.77(2); B1−Sn1−Sn2−B2 28.1; B3−Sn3−Sn4−B4 125.0.

Finally, we examined the behaviour of **2** in the presence of protic reagents, with a view to providing a route to the parent neutral cyclobutene analogue, and also as a probe of sites of electron density within the dianionic [Sn_4_{B(NDippCH)_2_}_4_]^2−^ framework. Reaction of **2** with benzoic acid in benzene solution leads to the formation of dark purple crystals of the doubly protonated product Sn_4_{B(NDippCH)_2_}_4_H_2_ (**4**) in good (ca. 60 %) yield. **4** has been characterized by NMR spectroscopy and X‐ray crystallography (Scheme 4 and Figure 5). The (single) hydride signal appears at 4.25 ppm with two sets of satellites being resolved due to coupling to the ^119^Sn and ^117^Sn nuclei (*J*_119Sn−H_=53.9 Hz, *J*_117Sn−H_=45.6 Hz). The solid state structure shows a short Sn(1)−Sn(2) distance (2.6737(7) Å) and three longer Sn−Sn distances (2.8045(7), 2.8232(5), 2.8333(5) Å), consistent with a structure featuring a formal Sn=Sn double bond and three single bonds, respectively (Figure 5). The B(1)Sn(1)Sn(2)B(2) unit approaches coplanarity (torsion angle=28.1°), while the B(3)Sn(3)Sn(4)B(4) unit features a much wider torsion angle of 125.0°. While the location of the tin‐bound hydrogen atoms by X‐ray crystallography must be viewed critically, the alignment of the boryl groups at Sn(3) and Sn(4) is consistent with the projection of the two Sn−H bonds either side of the Sn_4_ plane, and with the overall *C*_2_ symmetry implied by solution phase NMR measurements.

From a synthetic perspective, protonation in this (1,2‐*trans*) fashion is consistent (i) with a 1,2‐localization of negative charge in **2** in a manner similar to that proposed by Sekiguchi and co‐workers for Si_2_Ge_2_ system **III**,[^12^, ^13^] and (ii) with the steric bulk of the boryl substituents favouring an anticlinal rather than eclipsed conformation about the Sn(3)−Sn(4) bond. Consistently, Natural Resonance Theory (NRT) calculations show that the primary resonance contributions (>80 %) involve a Sn=Sn double bond and 1,2‐localized negative charges (Scheme 4); this model of electronic structure also provides a rationale for the non‐aromatic character of **2** determined by the NICS calculations.

## Conclusions

In conclusion, we have shown that the controlled reduction of the boryl‐substituted Sn^II^ precursor {(HCDippN)_2_B}Sn‐(IPrMe)Br with 1.5 equivalents of potassium graphite leads to the generation of the cyclic tetra‐tin tetra‐boryl system K_2_[Sn_4_{B(NDippCH)_2_}_4_], a homo‐metallic heavier analogue of the cyclobutadiene dianion. This system is non‐aromatic, with the primary contributing resonance structures involving a Sn=Sn double bond and 1,2‐localized negative charges. Attempted abstraction of the K^+^ cations or oxidation lead to contraction or cleavage of the Sn_4_ unit, respectively, while protonation generates the neutral dihydride 1,2‐Sn_4_{B(NDippCH)_2_}_4_H_2_ in a manner consistent with the predicted charge distribution. Interestingly, although the 6π‐system [Sn_4_{B(NDippCH)_2_}_4_]^2−^, and 2π‐electron systems [In_4_{B(NDippCH)_2_}_4_]^2−^ and (hypothetical) [Sn_4_{B(NDippCH)_2_}_4_]^2+^ conform to the Hückel 4*n*+2 stipulation for aromaticity, only the two‐electron systems appear to show aromatic character (for the [Sn_4_{B(NDippCH)_2_}_4_]^2+^ dication: NICS(0)=−9.46; NICS(1)=−11.76 (1)).^[17]^ Such a finding is consistent with the key electronic structure‐defining role predicted for enhanced Coulombic repulsions in 6π electron systems bearing a double *negative* charge.[^3^, ^18^]

## Experimental Section

Selected synthetic and characterizing data are given here. Complete experimental data, representative spectra, details of quantum chemical calculations and CIFs relating to the X‐ray crystal structures can be found in the Supporting Information.

**K_2_[Sn_4_{B(NDippCH)_2_}_4_] (2)**: A mixture of {(HCDippN)_2_B}Sn‐(IPrMe)Br (0.20 g, 0.26 mmol) and KC_8_ (0.055 g, 0.41 mmol) was dissolved/suspended in toluene (5 mL) at room temperature. The reaction mixture was stirred and monitored by NMR until most of the starting material turned into the desired product. The reaction mixture was then filtered into a Schlenk tube and concentrated. Pentane (5 mL) was then added and the tube was stored at 4 °C overnight to give black crystals suitable for X‐ray crystallography, which were isolated, washed with small amount of cold (−20 °C) pentane and dried in vacuo. Yield: 0.051 g, 36.4 %. Anal. Calc. for C_52_H_72_B_2_KN_4_Sn_2_: C 59.41 %, H 6.90 %, N 5.33 %; Meas.: C 59.58 %, H 7.27 %, N 5.34 %. ^1^H NMR (400 MHz, C_6_D_6_, 298 K): δ_H_ 0.64 (d, *J*_HH_=6.8 Hz, 12H, CH(C*H_3_*)_2_ of Dipp), 1.15 (d, *J*_HH_=6.8 Hz, 12H, CH(C*H_3_*)_2_ of Dipp), 1.39 (d, *J*_HH_=6.8 Hz, 12H, CH(C*H_3_*)_2_ of Dipp), 1.62 (d, *J*_HH_=6.8 Hz, 12H, CH(C*H_3_*)_2_ of Dipp), 3.45 (sept, *J*_HH_=6.8 Hz, 4H, C*H*(CH_3_)_2_ of Dipp), 3.60 (sept, *J*_HH_=6.8 Hz, 4H, C*H*(CH_3_)_2_ of Dipp), 6.04 and 6.36 (d, *J*_HH_=2.2 Hz, 4H, C*H* of boryl), 6.73–6.81 (m, 6H, Ar*H* of Dipp⋅⋅⋅K), 7.20–7.32 (m, 6H, Ar*H* of Dipp). ^11^B{^1^H} NMR (128 MHz, C_6_D_6_, 298 K): δ_B_ 49.4. ^13^C NMR (126 MHz, C_6_D_6_, 298 K) δ_C_ 24.8, 25.3, 25.8 and 26.2 (CH(*C* H_3_)_2_ of Dipp), 28.6 and 28.8 (*C* H(CH_3_)_2_ of Dipp), 122.6 (*C* H of boryl), 123.5 and 123.6 (*m*‐Ar of Dipp), 123.7 and 123.8 (*p*‐Ar of Dipp), 144.1 and 144.2 (*o*‐Ar of Dipp), 147.0 and 148.7 (*i‐*Ar of Dipp). UV‐Vis (methylcyclohexane): λ_max_=432 nm, ϵ=10,100 L mol^−1^ cm^−1^.

**Sn_4_{B(NDippCH)_2_}_4_H_2_ (4)**: To a mixture of **2** (20 mg, 0.019 mmol) and PhCO_2_H (2.3 mg, 0.019 mmol) was added benzene (5 mL). The solution was stirred for 10 min and dried under vacuum to remove all volatiles. The residues were then extracted into pentane and concentrated to about half of the original volume, stored at −30 °C to afford dark purple small crystals which were suitable for crystallography. Yield: 12 mg, 62.3 %. ^1^H NMR (500 MHz, C_6_D_6_, 298 K): δ_H_ 0.40 (d, *J*_HH_=6.8 Hz, 6H, CH(C*H_3_*)_2_ of Dipp), 0.57 (d, *J*_HH_=6.8 Hz, 6H, CH(C*H_3_*)_2_ of Dipp), 0.99 (d, *J*_HH_=6.8 Hz, 6H, CH(C*H_3_*)_2_ of Dipp), 1.10 (dd, *J*_HH_=6.8, 2.0 Hz, 12H, CH(C*H_3_*)_2_ of Dipp), 1.21 (m, 48H, CH(C*H_3_*)_2_ of Dipp), 1.31 (d, *J*_HH_=6.8 Hz, 6H, CH(C*H_3_*)_2_ of Dipp), 1.38 (dd, *J*_HH_=6.8, 2.0 Hz, 12H, CH(C*H_3_*)_2_ of Dipp), 2.53, 2.76, 2.78, 2.93, 3.09, 3.14, 3.31 and 3.48 (sept, *J*_HH_=6.8 Hz, 2H, C*H*(CH_3_)_2_ of Dipp), 4.25 (t, *J*_HSn119_=53.9 Hz, *J*_HSn117_=45.6 Hz, 2H, Sn*H*), 6.03, 6.15, 6.16 and 6.24 (d, *J*_HH_=2.0 Hz, 2H, C*H* of boryl), 6.85 (d, *J*_HH_=7.6 Hz, 2H, *p‐*Ar*H*), 7.07–7.19 (m, 6H, *p‐*Ar*H*), 7.21–7.35 (m, 16H, *m‐*Ar*H*). ^11^B{^1^H} NMR (128 MHz, C_6_D_6_, 298 K): δ_B_ 32.2 ([B]SnH), 44.9 ([B]Sn). ^13^C{^1^H} NMR (126 MHz, C_6_D_6_, 298 K): δ_C_ 23.8, 23.9, 24.4, 24.5, 25.0, 25.2, 25.4 and 25.6 (CH(*C* H_3_)_2_ of Dipp), 25.9, 26.2, 26.6, 26.7, 26.8, 27.0, 27.3, 27.5, 27.9, 27.9, 28.1, 28.4, 28.5, 28.6, 28.7 and 29.3, (*C* H(CH_3_)_2_ of Dipp), 119.8, 122.8, 123.1 and 123.4 (*C* H of boryl), 123.6, 123.7, 123.8, 123.9, (*p*‐Ar of Dipp), 124.0, 124.1, 124.2 and 125.1, (*m*‐Ar of Dipp), 139.8, 140.0, 140.2, 141.4, (*o*‐Ar of Dipp), 145.5, 145.7, 145.9, 146.2, 146.6, 146.8, 147.1 and 147.1 (*i*‐Ar of Dipp). UV‐Vis (methylcyclohexane): λ_max_=529 nm, ϵ=2,240 L mol^−1^ cm^−1^.

## Conflict of interest

The authors declare no conflict of interest.

1

## Supporting information

As a service to our authors and readers, this journal provides supporting information supplied by the authors. Such materials are peer reviewed and may be re‐organized for online delivery, but are not copy‐edited or typeset. Technical support issues arising from supporting information (other than missing files) should be addressed to the authors.

Supporting Information

## Data Availability

The data that support the findings of this study are available in the supplementary material of this article.
