# Supplementary material for: Synthesis of Homo‐Metallic Heavier Analogues of Cyclobutene and the Cyclobutadiene Dianion
Source: Chemistry. 2023 Feb 27;29(20):e202300006. doi: 10.1002/chem.202300006 (PMC10947146; doi:10.1002/chem.202300006)
Supplement: Supplementary file 1 — Supporting Information [file CHEM-29-0-s001.pdf]

Chemistry–A European Journal

Supporting Information

Synthesis of Homo-Metallic Heavier Analogues of Cyclobutene and the Cyclobutadiene Dianion

Xiongfei Zheng, Agamemnon E. Crumpton, Andrey V. Protchenko, Mathias A. Ellwanger, Andreas Heilmann, and Simon Aldridge*

1. Synthetic/characterizing data and NMR spectra of novel compounds s2

2. Details of DFT calculations and *xyz* files s8

3. References for supporting information s15

1. Synthetic/characterizing data and NMR spectra for novel compounds

$K_2[Sn_4\{B(NDippCH)_2\}_4]$ (2): A mixture of $\{(HCDippN)_2B\}Sn(IPrMe)Br$ (0.20 g, 0.26 mmol) and KC_8 (0.055 g, 0.41 mmol) was dissolved/suspended in toluene (5 mL) at room temperature. The reaction mixture was stirred and monitored by NMR until most of the starting material turned into the desired product. The reaction mixture was then filtered into a Schlenk tube and concentrated. Pentane (5 mL) was then added and the tube was stored at 4 °C overnight to give black crystals suitable for X-ray crystallography, which were isolated, washed with small amount of cold (-20 °C) pentane and dried in vacuo. Yield: 0.051 g, 36.4 %. Anal. Calc. for $C_{52}H_{72}B_2KN_4Sn_2$: C 59.41 %, H 6.90 %, N 5.33 %; Meas.: C 59.58 %, H 7.27 %, N 5.34 %.

^{1}H NMR (400 MHz, C_6D_6, 298 K): δ_H 0.64 (d, J_{HH} = 6.8 Hz, 12H, CH(C*H*$_3$)$_2$ of Dipp), 1.15 (d, J_{HH} = 6.8 Hz, 12H, CH(C*H*$_3$)$_2$ of Dipp), 1.39 (d, J_{HH} = 6.8 Hz, 12H, CH(C*H*$_3$)$_2$ of Dipp), 1.62 (d, J_{HH} = 6.8 Hz, 12H, CH(C*H*$_3$)$_2$ of Dipp), 3.45 (sept, J_{HH} = 6.8 Hz, 4H, C*H*(CH$_3$)$_2$ of Dipp), 3.60 (sept, J_{HH} = 6.8 Hz, 4H, C*H*(CH$_3$)$_2$ of Dipp), 6.04 and 6.36 (d, J_{HH} = 2.2 Hz, 4H, C*H* of boryl), 6.73-6.81 (m, 6H, Ar*H* of Dipp···K), 7.20-7.32 (m, 6H, Ar*H* of Dipp).

^{11}B{^{1}H} NMR (128 MHz, C_6D_6, 298 K): δ_B 49.4.

^{13}C NMR (126 MHz, C_6D_6, 298 K) δ_C 24.8, 25.3, 25.8 and 26.2 (CH(*C̲*H$_3$)$_2$ of Dipp), 28.6 and 28.8 (*C̲*H(CH$_3$)$_2$ of Dipp), 122.6 (*C̲*H of boryl), 123.5 and 123.6 (*m*-Ar of Dipp), 123.7 and 123.8 (*p*-Ar of Dipp), 144.1 and 144.2 (*o*-Ar of Dipp), 147.0 and 148.7 (*i*-Ar of Dipp).

UV-Vis (methylcyclohexane): λ_{max} = 432 nm, ε = 10,100 L mol^{-1} cm^{-1}.

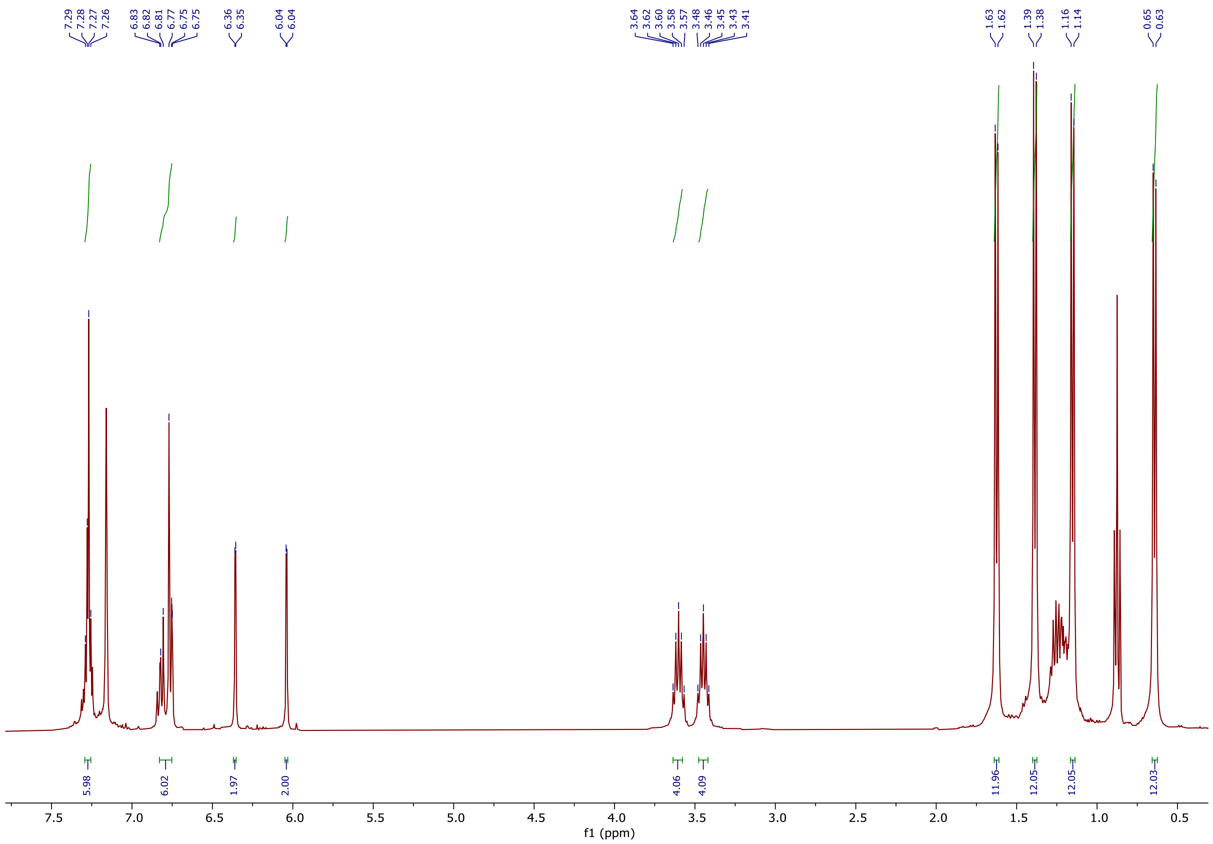
7.29
7.28
7.27
7.26
6.83
6.82
6.81
6.77
6.75
6.75
6.36
6.35
6.04
6.04
3.64
3.62
3.60
3.58
3.57
3.48
3.46
3.45
3.43
3.41
1.63
1.62
1.39
1.38
1.16
1.14
0.65
0.63
5.98
6.02
1.97
2.00
4.06
4.09
11.96
12.05
12.05
12.03
f1 (ppm)

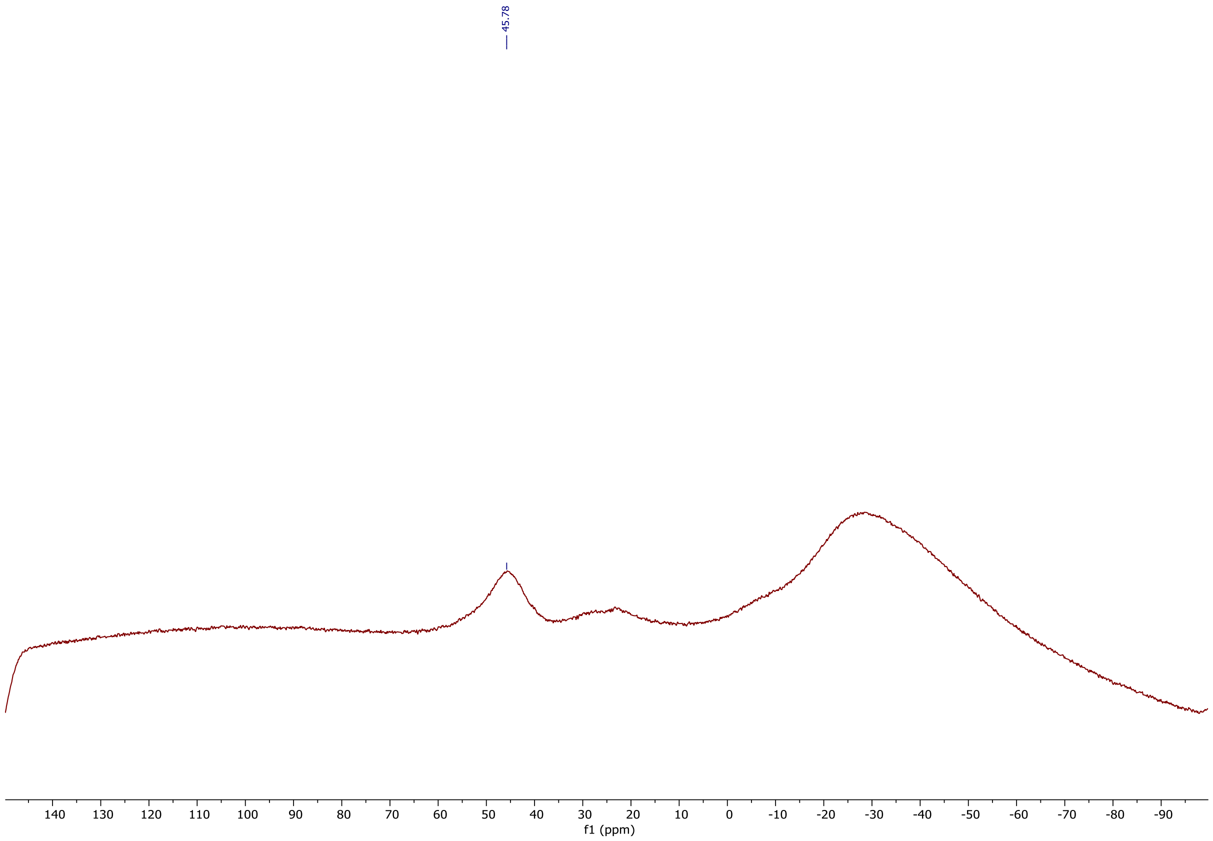
45.78
f1 (ppm)

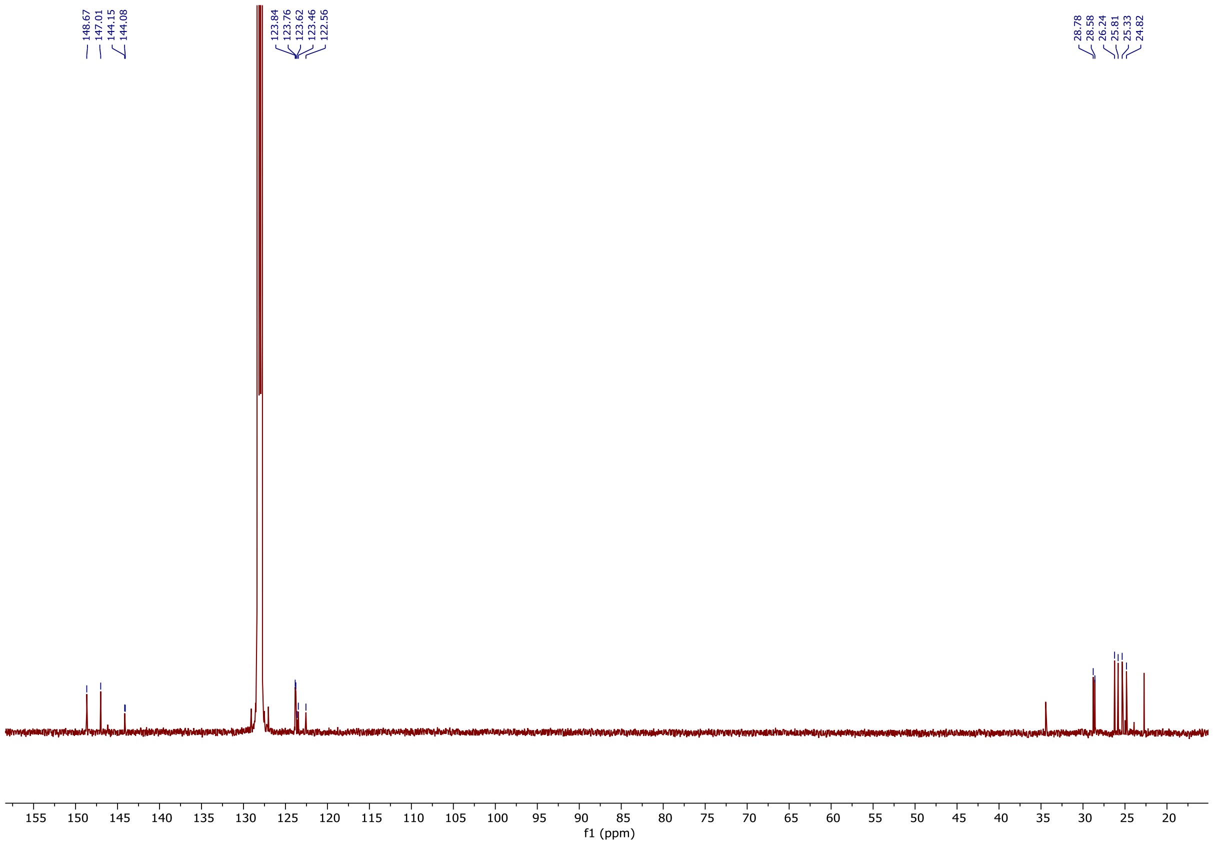

Figure s1: ^{1}H, $^{11}B\{^{1}H\}$ and $^{13}C\{^{1}H\}$ NMR spectra of **2** in C_6D_6 at 298 K.

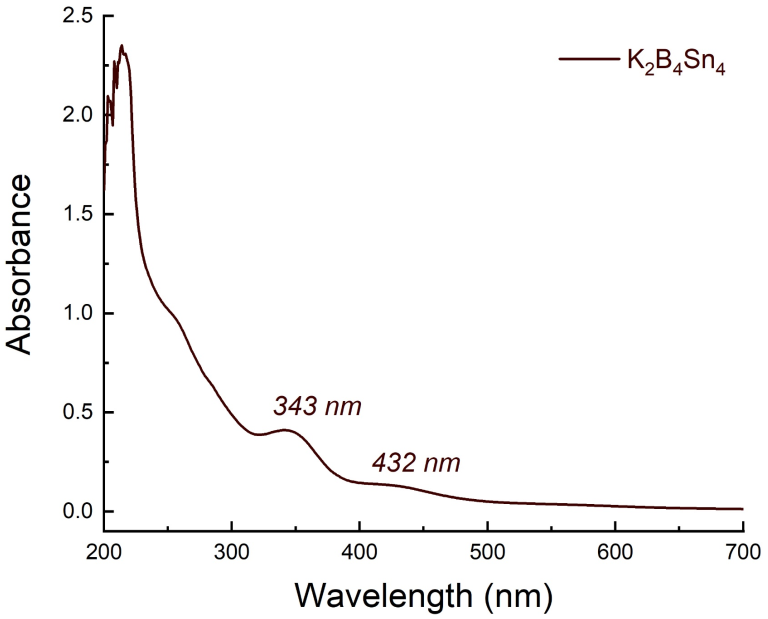

Figure s2: UV-Vis spectrum of **2** in methyl-cyclohexane at 298 K.

[K(2.2.2-crypt)]$_2$[Sn$_4${B(NDippCH)$_2$}$_2$] (3): To a mixture of **2** (5 mg, 0.002 mmol) and 2,2,2-cryptand (1.8 mg, 0.004 mmol) was added C_6D_6 (0.3 mL). The colour of the solution immediately turned pale and dark purple crystals precipitated, which were suitable for X-ray crystallography. The crystals were isolated, washed with a small amount of pentane and dried in vacuo. Yield: 2.5 mg, 50.5 %. **3** is resolutely insoluble in compatible solvents, so NMR spectra could not be obtained.

Sn$_4${B(NDippCH)$_2$}$_4$H$_2$ (4): To a mixture of **2** (20 mg, 0.019 mmol) and $PhCO_2H$ (2.3 mg, 0.019 mmol) was added benzene (5 mL). The solution was stirred for 10 min and dried under vacuum to remove all volatiles. The residues were then extracted into pentane and concentrated to about half of the original volume, stored at – 30 °C to afford dark purple small crystals which were suitable for crystallography. Yield: 12 mg, 62.3 %.

^{1}H NMR (500 MHz, C_6D_6, 298 K): δ_H 0.40 (d, J_{HH} = 6.8 Hz, 6H, CH(C*H$_3$*)$_2$ of Dipp), 0.57 (d, J_{HH} = 6.8 Hz, 6H, CH(C*H$_3$*)$_2$ of Dipp), 0.99 (d, J_{HH} = 6.8 Hz, 6H, CH(C*H$_3$*)$_2$ of Dipp), 1.10 (dd, J_{HH} = 6.8, 2.0 Hz, 12H, CH(C*H$_3$*)$_2$ of Dipp), 1.21 (m, 48H, CH(C*H$_3$*)$_2$ of Dipp), 1.31 (d, J_{HH} = 6.8 Hz, 6H, CH(C*H$_3$*)$_2$ of Dipp), 1.38 (dd, J_{HH} = 6.8, 2.0 Hz, 12H, CH(C*H$_3$*)$_2$ of Dipp), 2.53, 2.76, 2.78, 2.93, 3.09, 3.14, 3.31 and 3.48 (sept, J_{HH} = 6.8 Hz, 2H, C*H*(CH$_3$)$_2$ of Dipp), 4.25 (t, J_{HSn119} = 53.9 Hz, J_{HSn117} = 45.6 Hz, 2H, Sn*H*), 6.03, 6.15, 6.16 and 6.24 (d, J_{HH} = 2.0 Hz, 2H, C*H* of boryl), 6.85 (d, J_{HH} = 7.6 Hz, 2H, *p*-Ar*H*), 7.07-7.19 (m, 6H, *p*-Ar*H*), 7.21-7.35 (m, 16H, *m*-Ar*H*).

^{11}B{^{1}H} NMR (128 MHz, C_6D_6, 298 K): δ_B 32.2 ([B]SnH), 44.9 ([B]Sn).

^{13}C{^{1}H} NMR (126 MHz, C_6D_6, 298 K): δ_C 23.8, 23.9, 24.4, 24.5, 25.0, 25.2, 25.4 and 25.6 (CH($\underline{C}$H$_3$)$_2$ of Dipp), 25.9, 26.2, 26.6, 26.7, 26.8, 27.0, 27.3, 27.5, 27.9, 27.9, 28.1, 28.4, 28.5, 28.6, 28.7 and 29.3, ($\underline{C}$H(CH$_3$)$_2$ of Dipp), 119.8, 122.8, 123.1 and 123.4 ($\underline{C}$H of boryl), 123.6, 123.7, 123.8, 123.9, (*p*-Ar of Dipp), 124.0, 124.1, 124.2 and 125.1 , (*m*-Ar of Dipp), 139.8, 140.0, 140.2, 141.4, (*o*-Ar of Dipp), 145.5, 145.7, 145.9, 146.2, 146.6, 146.8, 147.1 and 147.1 (*i*-Ar of Dipp).

UV-Vis (methylcyclohexane): λ_{max} = 529 nm, ε = 2,240 L mol^{-1} cm^{-1}.

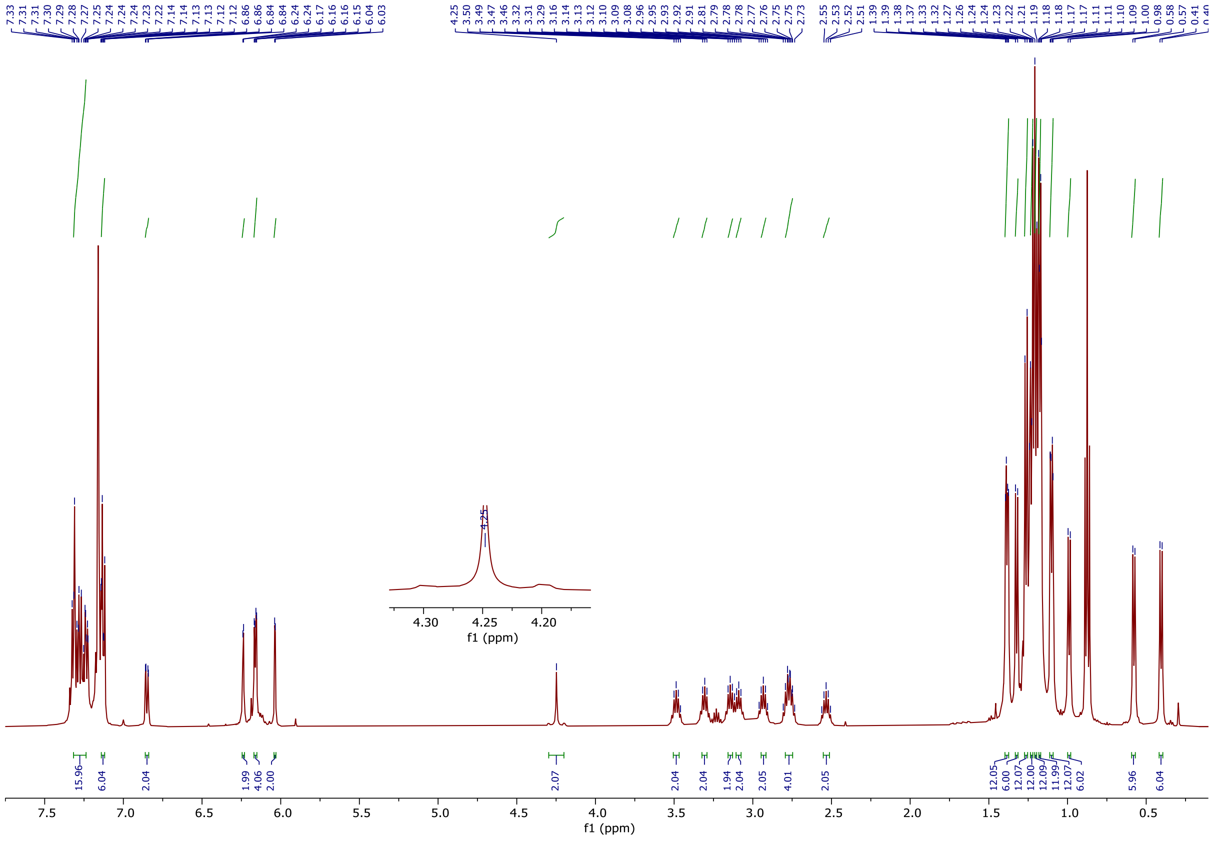

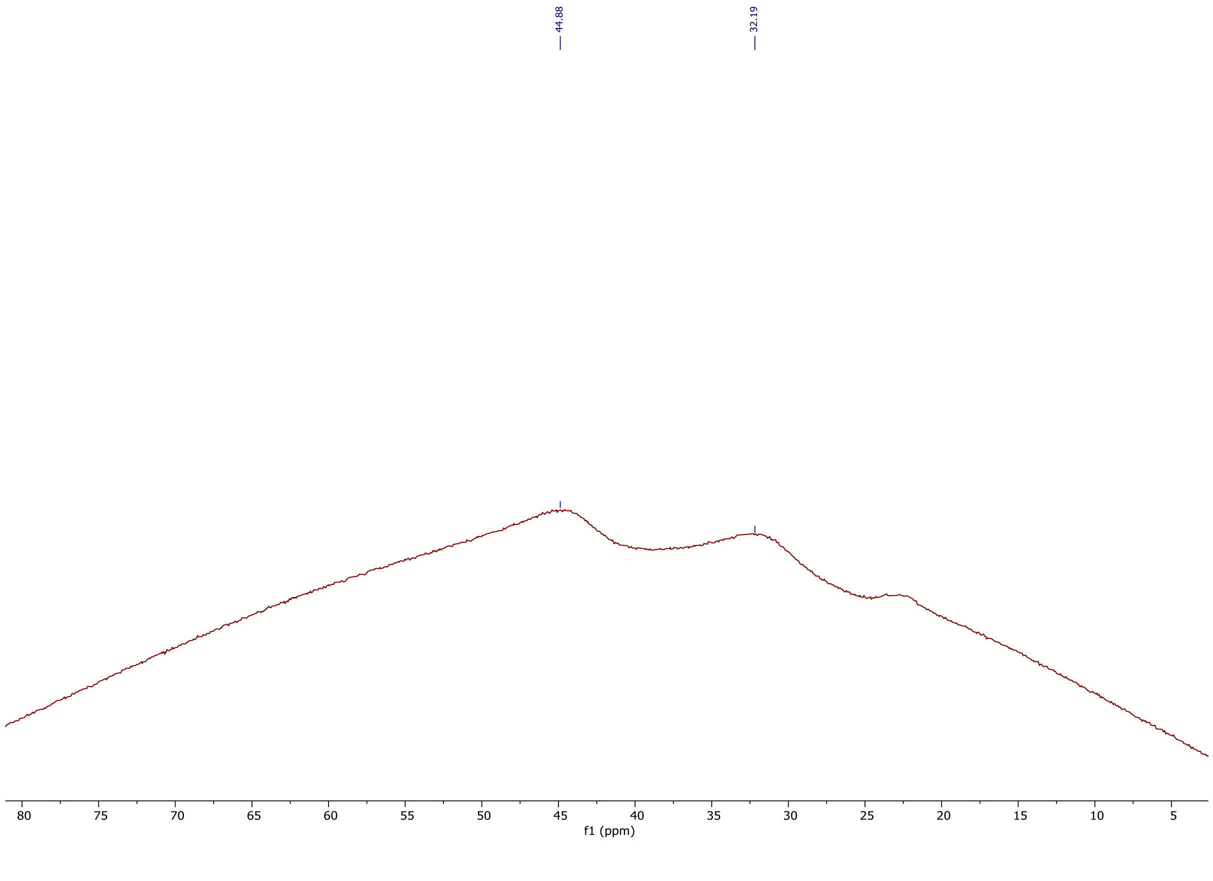

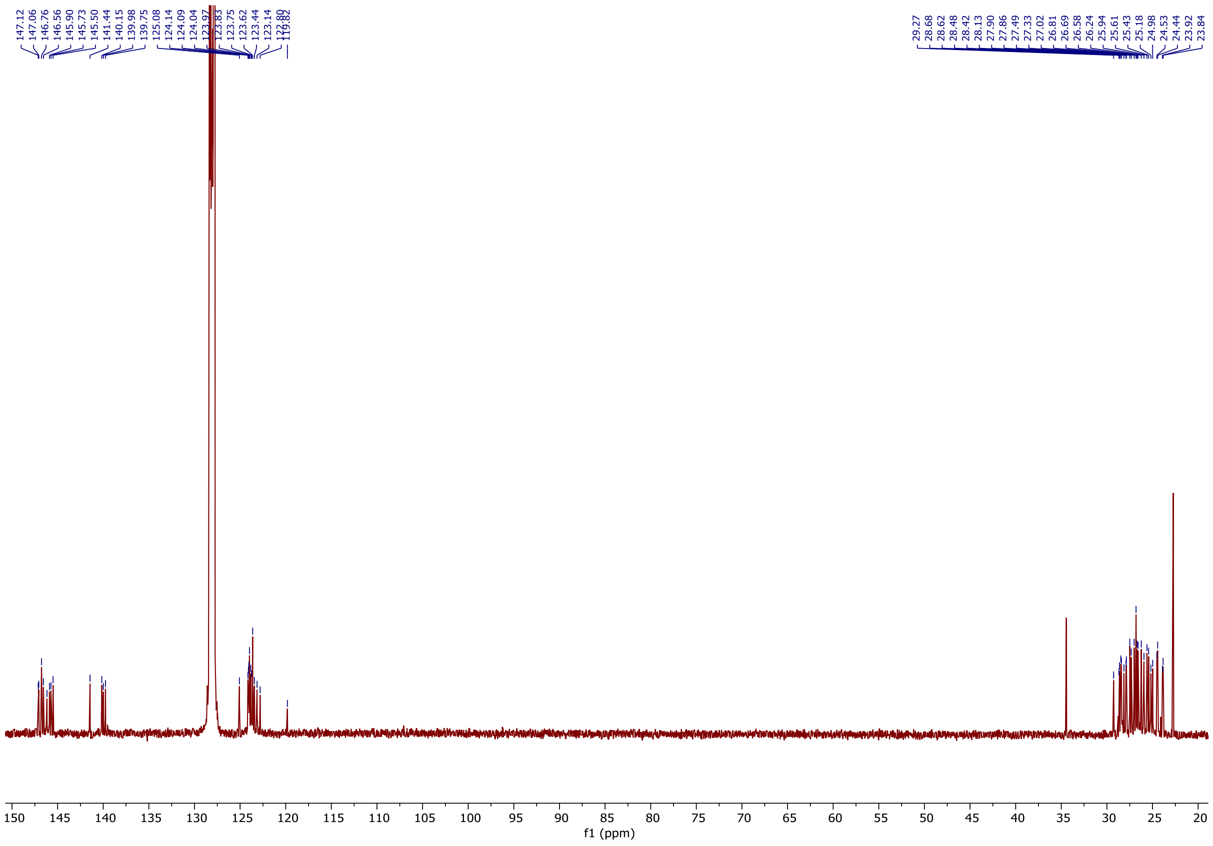

Figure s3: ^{1}H, $^{11}B\{^{1}H\}$ and $^{13}C\{^{1}H\}$ NMR spectra of **4** in C_6D_6 at 298 K.

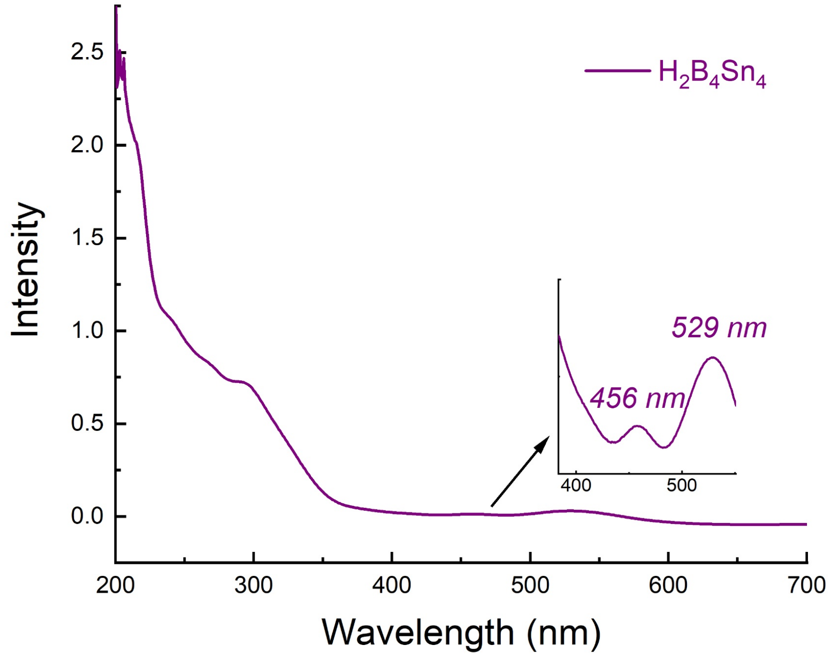

Figure s4: UV-Vis spectrum of **4** in methyl-cyclohexane at 298 K.

2. Details of DFT calculations and *xyz* files

General details: All computational work reported here was performed using ORCA (Revision 5.0.3).[s1] The meta-generalized-gradient approximation (mGGA) functional R2-SCAN[s2] was employed in conjunction with the Def2-TZVPPm[s3] basis set with the D4 dispersion correction,[s4] and employing the geometrical counterpoise correction gCP[s5] (together known as the R2SCAN-3c method).[s3] The nature of the stationary points (minima) was confirmed by full frequency calculations, and are characterized by zero imaginary frequencies.

Aromaticity indices: Nuclear independent chemical shift (NICS)[s6] calculations where performed with the R2-SCAN functional in conjunction with the Def2-TZVP basis set and D4 dispersion correction.[s7] Ring Critical Point (RCP) properties;[s8] electron density (ρ) and curvature perpendicular to the ring ($\lambda_{\sigma3}$), and Multicentre Index (MCI) calculations [s9] were carried out using the optimised electronic structure from the R2-SCAN-3C method using the Multiwfn program.[s10] RCP properties are strictly not comparable between rings of different elements however the Sn rings can all be compared to one another.[s11] Larger values of ρ and negative $\lambda_{\sigma3}$ have been shown to correspond to aromatic species. MCI is necessarily ring size depended however has been normalised such that it is comparable to benzene by the expression $-|MCBO|^{1/n}$.[s12]

Table s1: Aromaticity indices

	NICS(ppm)		RCP		MCI	
	0	1	ρ (e Å^{-3})	$\lambda_{\sigma3}$ (e Å^{-5})	MCI	Normalised MCI
K_2Sn_4	-3.167	-0.275	0.091729116	0.023823206	-0.0282983972	-0.4101478691
K_2In_4	-7.523	-8.608	0.096298004	-0.086809507	0.0601362506	0.4952039347
$[Sn_4]^{2+}$	-9.460	-11.760	0.095304494	-0.10364191	0.0153103166	0.3517596679
H_2Sn_4	-4.989	-4.826	0.097114984	-0.036569265	0.0040599670	0.2524239897
C_6H_6	-7.813	-9.813	0.137790173	-0.404925105	0.0823792309	0.6596338827

CASSCF calculations: The small HOMO-LUMO gap (0.8454 eV) calculated using the R2-SCAN-3c method led us to explore multi-configurational methods to probe further the electronic structure of **2**. CASSCF (6,4) was performed on the Hückel type *p* orbitals based on the natural MP2 orbitals of system **2** using the geometry from the R2-SCAN-3c method. Orbitals **A**-**D** show the CASSCF orbitals; significant population of the complete out-of-phase orbital D (1.32 e) was found with 15.3% of the ground state seeing this orbital filled. The CASSF "HOMO-LUMO gap" (the difference in energy between **C** and **D**) is 1.2898 eV.

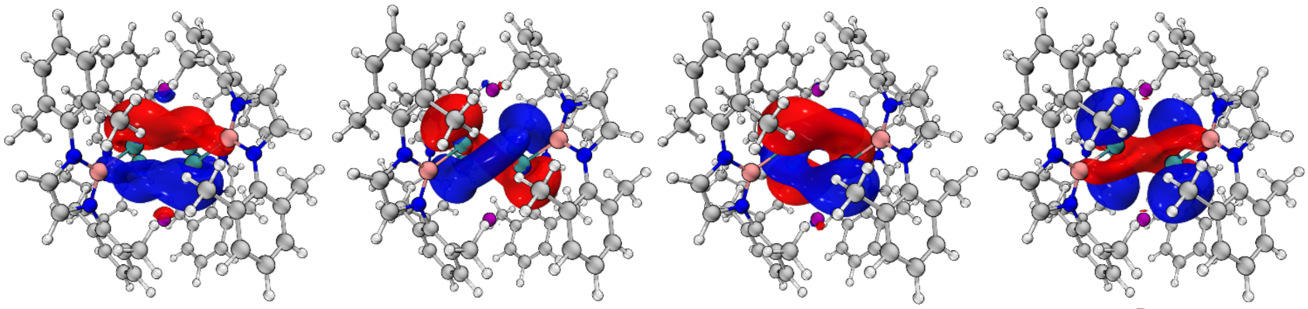

A **B** **C** **D**

Figure s5: CASSF active orbitals with iso-values of 0.02.

Table s2: Density matrix.

	A	B	C	D
A	1.759837	0	0	0
B	0	1.513581	0	0
C	0	0	1.399572	0
D	0	0	0	1.327010

Table s3: Root 0 composition.

%	A	B	C	D
84.352	2	2	2	0
8.115	2	2	0	2
7.185	2	0	2	2

NRT calculations: Natural resonance theory calculations were performed using the NBO7 program,[s13] using the R2-SCAN-3c methodology. A model system was used to allow convergence of the NRT module (in which the B-bound Dipp groups were replaced by methyl groups) and employing a constrained optimisation fixing the K, Sn, B, N positions.

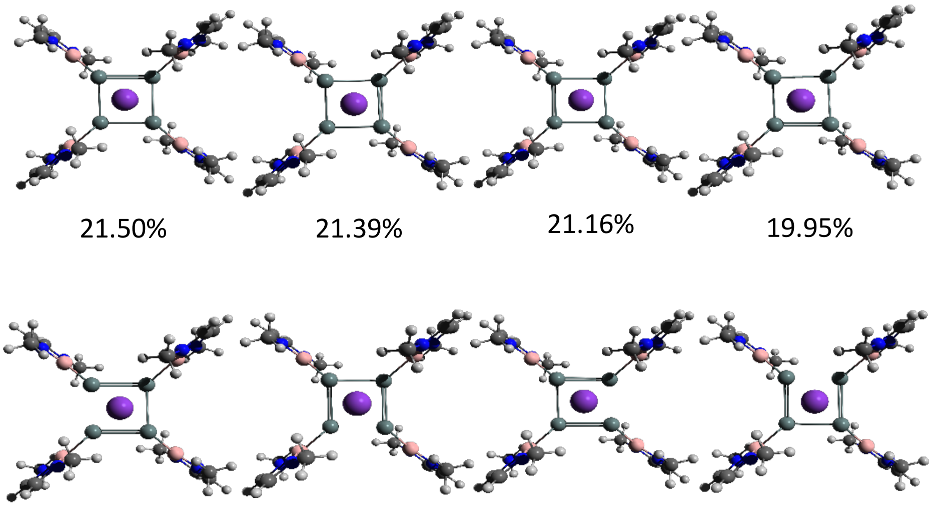

21.50% 21.39% 21.16% 19.95%

4.13% 4.00% 3.96% 3.90%

Figure s6: Composition of the NRT resonance forms and their relative contributions.

Xyz coordinates:

```
K2Sn4
170
  Sn  7.93290493774198     9.73271962052571     18.18778129875407
  K   6.55538705752630    11.10118616115493     21.89885676172339
  N   9.65119567899513     8.15392890370708     20.70980679020758
  N   10.08363214126653     7.29055081597023     18.66449688921043
  C   9.24570197438354     8.87361163358544     21.86735545419220
  C   7.94439297329200     8.99798212117212     23.89211053946865
  H   7.19515312906388     8.57465203412297     24.55731096325445
  C   10.20561128595172     7.04604744906534     17.26718274888351
  C   8.25845311554722     8.32695439237942     22.70535632901593
  C   9.88932063563281    10.08090893232666     22.18515168958052
  C   8.58109105051099    10.19109500438132     24.22673290315983
  H   8.33585525476881    10.69555203329889     25.15793542137581
  C   10.57074041471174     7.10123862113196     20.83904186475149
  H   10.96577103352032     6.80488558980687     21.80130155644716
  C   10.83166235125445     6.59007876158855     19.61335767222666
  H   11.48907280647833     5.77552810382910     19.34105111819133
  C   9.22847511619242     6.28075749134842     16.61723778020956
  C   9.53354221508580    10.73485301015907     23.36967127655556
  H   10.02520560875197    11.67102276322254     23.62279985375789
  C   9.35379247130366     6.07779507625708     15.24200594213429
  H   8.60758452393049     5.47902981580519     14.72568033711258
  C   11.29284089745260     7.59609481335682     16.57391820886884
  C   10.41997406900082     6.62236438770293     14.53586184715080
  H   10.50645867735147     6.45175134611652     13.46616736656875
  B   9.29989470732538     8.31995109715455     19.31253941806798
  C   11.38280130628466     7.37514591969999     15.19872137667639
  H   12.22013611522758     7.79582129789522     14.64658811068746
  Sn  5.20635617640549    12.46571575581121     18.15108044717714
  N   3.46598805249723    14.04753346047258     20.66023093726620
  N   3.04829727306423    14.90244071905149     18.60850438684803
  C   3.86447354354554    13.33499897755861     21.82460376741167
  C   5.15329249801378    13.22421459998382     23.85802846605053
  H   5.89911070675959    13.65154171414895     24.52452902385714
  C   2.93899549050556    15.14303385161530     17.20948344909741
  C   4.84790850269641    13.88630439525404     22.66397876701810
  C   3.21672828198215    12.13139913028434     22.14783086935367
  C   4.51195820008496    12.03520055726502     24.19833279882641
  H   4.75012028222280    11.53795891516800     25.13525295811788
  C   2.54341805625791    15.09894591741211     20.77817794982059
  H   2.14048820861069    15.39830819817715     21.73622569246833
  C   2.29148258180358    15.60519253325662     19.54858983175581
  H   1.63505337921313    16.41775339121033     19.26807806530547
  C   3.92143528123534    15.90761794052242     16.56671521703585
  C   3.56395088651060    11.48621027989885     23.33965984712306
  H   3.06894751255943    10.55301185541214     23.59721764754273
  C   3.80908480384354    16.10646797593094     15.18977981952791
  H   4.55952212138572    16.70452726128849     14.67877782082000
  C   1.85893094228590    14.58996318912715     16.50756248339050
  C   2.75022137031450    15.55871656391284     14.47511863332512
  H   2.67373650233790    15.72616664989195     13.40416519798052
  B   3.82901325140676    13.87688401319081     19.26652789537854
  C   1.78177426146328    14.80697815837765     15.13097281513883
  H   0.95036583306150    14.38348111474403     14.57207470731799
  Sn  5.19751326911230     9.73825051926593     18.99434058344915
  K   6.58463498649057    11.08094002513514     15.27029217304906
  N   3.48031890790762     8.14864747012671     16.47648324437391
  N   3.04723867722332     7.29537476477334     18.52585147996080
  C   3.88645785838575     8.86219736000153     15.31538183473233
  C   5.18670121592801     8.97441183665107     13.28925019833198
  H   5.93459394766671     8.54641681611175     12.62550550097431
  C   2.92498771937999     7.05760228494737     19.92431279322856
  C   4.87208971078499     8.30962848829626     14.47936909659201
  C   3.24471757633126    10.06903647752886     14.99209434511856
  C   4.55223323527876    10.16727840104439     12.94947997374642
  H   4.79796565199773    10.66692563041226     12.01581618737613
  C   2.55992281721147     7.09607489710478     16.35229671523271
  H   2.16448557347769     6.79560613691278     15.39148002637680
  C   2.29854033465170     6.59103226129642     17.58041412830273
  H   1.64031592629220     5.77845278048193     17.85663044730894
  C   3.90055378577199     6.29327527555685     20.57769322162869
  C   3.60128193317231    10.71686677640347     13.80446272623154
  H   3.11127305207034    11.65278732577750     13.54726093991565
  C   3.77479312080435     6.09671585238248     21.95381570140036
  H   4.51982349121799     5.49882784032701     22.47285824644642
  C   1.83911258074575     7.61330397787142     20.61516123707572
  C   2.70985654680153     6.64681391092581     22.65753257096094
  H   2.62304194583899     6.48121787989943     23.72798662150985
  B   3.83214492035962     8.32082513282077     17.87291142227571
  C   1.74869302061805     7.39877960339301     21.99132762848078
  H   0.91249310369427     7.82405875271774     22.54164180005884
  Sn  7.92994639016056    12.46634224749567     19.00512081162340
  N   9.67961694517712    14.02694827007162     16.49079193919519
  N   10.09963742785970    14.89178580554015     18.53791451054541
  C   9.27927589386533    13.30894326411253     15.33039319449301
  C   7.99082487858211    13.19015590840380     13.29716952139572
  H   7.24649551063465    13.61580635412936     12.62795083911674
  C   10.20795167034763    15.14095272123887     19.93550451936520
  C   8.29797390816101    13.85842825022017     14.48730494042119
  C   9.92341407101254    12.10147605200330     15.01444165036533
  C   8.62852074601442    11.99713635661801     12.96412584616797
  H   8.38903999207939    11.49508251423762     12.03011458709513
  C   10.60645329759537    15.07396979275588     16.36740117613589
  H   11.01113005645509    15.36624504564188     15.40790436992546
  C   10.85965055267236    15.58624990962718     17.59423907914663
  H   11.51912795406303    16.39778874589231     17.87052005251990
  C   9.22700861026624    15.91269442522545     20.57204113022595
  C   9.57455911926718    11.45029928513836     13.82633391928630
  H   10.06665350898085    10.51398071084861     13.57454241179443
  C   9.33822678945645    16.12016037359584     21.94779539882054
  H   8.58887325393810    16.72364786667739     22.45397945986310
  C   11.28530389649781    14.58896244839027     20.64239858041060
  C   10.39435496210857    15.57358635714035     22.66739332032506
  H   10.46984908100193    15.74751305768402     23.73738632548709
  B   9.31559223294466    13.86541587789303     17.88528999522909
  C   11.36117748918192    14.81442955561095     22.01771739904379
  H   12.19052333634370    14.39194242260807     22.58043638100229
  C   10.92821478903599    10.65521591420713     21.27084262014673
  H   11.40194728197891    11.52947355971968     21.72363107916589
  H   11.69394099960468     9.91121482943836     21.02553130271946
  H   10.46078173796344    10.96823860850564     20.32733872041858
  C   7.52959832536821     7.07713565677202     22.30754118372709
  H   6.92605311211260     7.26800094129308     21.41069987898338
  H   8.22055266609638     6.26604946380485     22.05675506789103
  H   6.86283775804607     6.74516366914865     23.10722431648160
  C   8.07241480673514     5.71057434844224     17.38732981856886
  H   7.47370950593278     5.04574562812184     16.75861357481751
  H   8.41383686977616     5.15461271748835     18.26709254170528
  H   7.42358217658567     6.51510241481617     17.75683432615314
  C   12.31896708470405     8.41900117329443     17.29982573173661
  H   11.83907389115674     9.21607435864195     17.87940172528969
  H   12.88676921619321     7.81267470383892     18.01464140794046
  H   13.02528089470004     8.87052686525957     16.59818025316330
  C   12.31586268332101    13.75944172837713     19.93047885146799
  H   13.01342117675240    13.30858275064241     20.64126470043777
  H   11.83894480024297    12.96138150433692     19.34980644772404
  H   12.89311589639812    14.36040557272379     19.21871328777255
  C   8.08146292037791    16.48393504485927     19.78714598781126
  H   8.43494445511053    17.03499226106264     18.90906014720010
  H   7.43406948548459    15.68018960076119     19.41348494442120
  H   7.47802912455937    17.15338396228545     20.40637199558342
  C   10.95705892855151    11.52534282901668     15.93360181291762
  H   10.48472899924135    11.21338225748407     16.87499044693633
  H   11.43132836880519    10.65014914985603     15.48313906428551
  H   11.72302533356851    12.26797167214003     16.18227788081810
  C   7.56863806782428    15.10859670117458     14.88299729297210
  H   6.90618349573928    15.44248363859669     14.08053516527248
  H   6.96053288991927    14.91704347987344     15.77660668516306
  H   8.25957944656294    15.91836875578593     15.13815094973585
  C   2.20605430574368    10.64853241473814     15.90337351947618
  H   1.73421221685851    11.52200354973830     15.44707434680389
  H   1.43882889665320     9.90663775441952     16.15042805372010
  H   2.67304108063415    10.96400683909491     16.84623816872770
  C   5.59846600067440     7.06005441113941     14.88253944444428
  H   6.20254585243050     7.25332507310252     15.77850112465120
  H   4.90580801173864     6.25155053051502     15.13696093211754
  H   6.26427665847894     6.72309784380081     14.08414326696644
  C   0.81499338286678     8.43546095573093     19.88557812056697
  H   1.29675225837729     9.23113313253562     19.30560983640700
  H   0.24833167956684     7.82823429717249     19.17063798659666
  H   0.10755883688501     8.88881895886497     20.58491753084138
  C   5.05600682124776     5.71808932100337     19.81036713134497
  H   5.65386118589942     5.05553897905034     20.44228693923738
  H   4.71412548436212     5.15841630103712     18.93313455491217
  H   5.70579734762684     6.52019025808016     19.43730179761660
  C   5.06939849337215    16.48108484266580     17.34647282166459
  H   4.71854631518189    17.04033411611859     18.22044310068858
  H   5.71427906125812    15.67802034653996     17.72592940583565
  H   5.67469816450983    17.14351422513993     16.72155587304001
  C   0.82694602376074    13.76798650707036     17.22614929960018
  H   0.12504391103700    13.31741671844684     16.51946320306741
  H   1.30227254301681    12.97006357293337     17.80835583930453
  H   0.25490012063756    14.37436148665161     17.93749101599316
  C   5.58086845431731    15.13190251263599     22.26064130547905
  H   6.24530504228454    15.46794979799910     23.06055422985045
  H   6.18729925866437    14.93385806397382     21.36727131688000
  H   4.89219960657975    15.94254343505759     22.00207993413564
  C   2.18127221862281    11.55304622149238     21.23210293125226
  H   2.65153436943319    11.23871434446468     20.29054406739872
  H   1.70748315000175    10.67919394980688     21.68570078641460
  H   1.41501939931308    12.29534015849020     20.98316950917249
K2In4
170
  C   6.28115026256370    14.54404849704408     28.58741463348538
  C   6.57359545658756    14.08160538945904     29.82689872067321
  C   4.80664173849136    16.23251384060921     27.56615768728112
  C   3.75118079873424    15.73664051869435     26.78315192458288
  C   3.35344897426666    16.46955255097835     25.65959545804388
  C   3.98160758115186    17.66925883317265     25.33333387478793
  C   5.00681791396681    18.16176340838807     26.13661865852045
  C   5.43631421336683    17.45227425061482     27.26314578582309
  H   3.15198676540511    19.52034786481947     38.14545738975712
  H   5.13862415638764    18.44058003954120     37.12818812736147
  H   -2.30635453693179    22.80425204736914     27.63650602234248
  B   5.09089416060486    21.23879884765680     32.39567755498059
  N   5.92701053333644    22.19342952400852     31.70353077717017
  N   5.32345883591614    21.57042062350053     33.78556441417457
  C   5.84961971969858    14.52388534536810     32.15956572667064
  C   6.71440373786321    15.34728980554797     32.89561349740760
  C   6.79135974723865    15.15985018831869     34.27677850786009
  C   6.02635616500868    14.18456804539458     34.90634593976333
  C   5.16577893674402    13.38630871952604     34.16201826598905
  C   5.05899861108715    13.54699961858134     32.77943225058773
  C   -0.39537276262349    20.84469564549947     27.56528156123187
  C   0.66196440214977    21.33927876563291     26.78396736722225
  In  3.40848418687344    16.98649460279826     31.17994198454233
  B   4.86623388612574    15.65048068096814     30.07903263251409
  N   5.74269849291299    14.71912079515739     30.75289906372462
  N   5.25032000450202    15.49187494413878     28.69166519442476
  K   2.20176651676095    18.53680868085743     27.93980277048825
  C   6.08335619866798    22.32773252019234     30.29467148233159
  C   -1.86898346067299    22.53267729248818     28.58785123128148
  C   6.36814144940290    22.55604287327776     27.54464593268754
  C   7.27715080518882    21.80026919215159     28.27622002845614
  H   6.71926205317973    14.27380713533324     27.63603012774293
  H   7.30652067918952    13.34240572707055     30.12165656036715
  H   2.54852364628981    16.08927613678422     25.03479306320573
  H   3.67105141552248    18.22120921395748     24.45013603269663
  H   5.48915006253840    19.10453988914949     25.88959620612180
  H   -2.89438931052618    23.73341357930232     30.12274872881678
  H   1.86547803419958    20.98636638128659     25.03630827451336
  H   8.10355022020341    21.30465723479321     27.77225976208465
  H   6.48773633381058    22.65733777033191     26.46918276237400
  H   4.59865846840623    23.77766117425428     27.61614108011194
  C   7.14456299161917    21.66760565630626     29.65941812819904
  In  1.00067456988854    20.08610594231911     31.18012550403945
  B   -0.45544127128929    21.42387122752108     30.07910298242119
  N   -1.33193542219868    22.35489028477491     30.75341439485775
  H   0.73865266227549    18.85784636993369     24.44739821913053
  H   -1.08259637935220    17.97644451102139     25.88415254719194
  H   6.53559124845580    23.05412135812700     34.82748636214605
  H   7.27031646297853    23.79287488316588     32.31674209431606
  H   2.13491432638530    21.51234029211621     37.07296115149499
  H   7.46348655833054    15.78492378841639     34.85956644624377
  H   6.10523979200107    14.04255190154086     35.98095321447224
  H   4.56725460696257    12.62351918629449     34.65414334616798
  C   -1.44009527017764    22.54797513780051     32.16028764475837
```

```
C   -2.30604353436497    21.72396298475773    32.89427600047372
C   1.05891881583137     20.60712752131742    25.65964098908404
C   0.42835842203740     19.40933547910865    25.33096435136458
C   -0.59880442069347    18.91811977970354    26.13254513367923
C   -2.16181461733289    22.99400284902374    29.82764471434745
C   5.31056686667057     23.18770399781534    28.18837570809271
C   5.15002602821957     23.08472418366265    29.57175760820522
C   4.70138697069230     19.32204509995759    36.66509969620908
C   -1.02761774750016    19.62687098236401    27.25977833802853
N   -0.83840465790044    21.58446223598886    28.69159640979039
C   3.57395380929041     21.60038820078767    35.47803969887106
C   -1.81602107713157    14.42302459246131    33.86661382383171
C   -2.18007786753456    14.05820917868406    32.61365194690770
C   -0.31925046794000    16.06740134224081    34.93089213690632
C   0.83040894767284     15.46691258945940    35.47286267295802
C   1.38647940372672     16.01762480944393    36.63228255366613
C   0.81878904807589     17.13990113104927    37.23060556335940
C   -0.29977194365459    17.74312908288124    36.66158179744825
C   -0.88377896037151    17.21976439415645    35.50286292961272
C   5.28688832572412     19.84636777572427    35.50758507702047
C   -2.38428134875949    21.90944386081549    34.27562745763695
C   -1.61940951312978    22.88342590249225    34.90737903907709
C   3.01648684768705     21.04876164202765    36.63634973562814
C   3.58275940721769     19.92537544835736    37.23390192503068
C   -1.67245031252044    14.74590828506680    30.28549922568747
C   -2.73184601839686    15.40706498453472    29.64836345136450
C   -2.86189313981380    15.27477856654514    28.26486682156030
C   -1.95248953749881    14.51789587502527    27.53494722272237
C   -0.89682779310268    13.88498356552190    28.18060816163643
C   -0.73866808604687    13.98786360581019    29.56424494497947
H   -1.69952317509569    23.02413033683246    35.98206699670254
C   -0.64983590479691    23.52379464749829    32.78230637875281
In  0.64986756423144     17.33123220569564    31.34923593122708
B   -0.68353490814377    15.83228652468252    32.38962665338035
N   -1.51900991029458    14.87900910386477    31.69480337501246
N   -0.91737014443279    15.49795985080289    33.77860061542370
K   2.20188318255829     18.53417223707427    34.60854300699179
H   -3.68698553633759    15.77104952964802    27.75941225817002
H   -2.06984903928764    14.41733914182750    26.45916939241032
H   -0.18469913854891    13.29400634041289    27.60971343323163
H   -3.05752646789856    21.28404528316324    34.85678139983492
H   -2.12931677395659    14.01133163576563    34.81654786538559
H   -2.86095633277532    13.27676877797133    32.30365395455379
H   2.26786061884330     15.55409544227096    37.06932056724742
H   1.24809128845166     17.54382141531192    38.14335708838206
H   -0.73830871979213    18.62345789418742    37.12558654788826
In  3.75906259097985     19.74104441683457    31.35184749880076
C   -0.75784424685286    23.68246024850944    34.16503088803330
C   6.22312823518546     22.64428268064349    33.87647556392129
H   -0.15985760462143    24.44462581228841    34.65876450330880
C   6.58876222161054     23.01117423875404    32.62458790105640
C   4.72382604225619     20.99990669945570    34.93653906420590
C   8.08533028365614     20.80663139496764    30.45337460177796
H   7.53221381850047     20.00790936909650    30.96346299637801
H   8.83857294577368     20.35134047893068    29.80481235840159
H   8.59679387219452     21.37943744029595    31.23470164184829
C   3.98954043313892     23.73591623600347    30.26812685623963
H   3.28315410832392     22.97113804629210    30.62110958174736
H   4.31169661327698     24.30013446280548    31.14960294565297
H   3.45830472327621     24.41091523378664    29.59087763294104
C   6.52303880972937     17.98275278436983    28.15038630695570
H   6.11276100725633     18.23024940734216    29.13944862781623
H   7.30506394675787     17.23295716901379    28.31065556733142
H   6.96705487278510     18.88716353487922    27.72778981057563
C   3.05658079535401     14.46308043843732    27.17149670244439
H   2.31718764457940     14.17809887053020    26.41852371230574
H   3.77067127779785     13.64304757565793    27.29852220533572
H   2.54625606479189     14.58496353935794    28.13591382053418
C   7.51018746524420     16.42039703799328    32.20889753910880
H   8.19312764275383     16.90828386094944    32.91001302450118
H   8.09199914030278     16.01537462979180    31.37395097642827
H   6.84045347675159     17.18226113099111    31.78672166457034
C   4.09921943858389     12.72248839877251    31.97069084350300
H   4.60500425908959     12.20267267016563    31.14986282755421
H   3.59691702398900     11.98158307616870    32.59856631896223
H   3.33780923244589     13.36552205342838    31.51208812416444
C   6.47683937704477     19.18942956507524    34.87127603532964
H   6.86742358036793     18.39118060934322    35.50753843938134
H   6.19757260022598     18.75760404964523    33.90133006016755
H   7.27206572483796     19.91548850036093    34.67405806674241
C   2.95351766446436     22.78795767527258    34.80212300158865
H   2.64937502057293     22.52703127084779    33.77964399033343
H   2.07159707564039     23.12909252034734    35.34922378701558
H   3.66644107042602     23.61519728717761    34.71642183147359
C   -3.10168943799041    20.65222742285929    32.20523271262733
H   -3.68195380639770    21.05859865242465    31.36987326341616
H   -2.43191127083950    19.89025869663238    31.78331052649428
H   -3.78613054855123    20.16425416215893    32.90482070061851
C   0.31149709857601     24.34870676661050    31.97582116182156
H   0.81299700279403     25.08889810467265    32.60516948777003
H   1.07347943636007     23.70586338027600    31.51786441183307
H   -0.19278798702445    24.86934806177388    31.15460568646298
C   -2.11599607700358    19.09729962016268    28.14557475554576
H   -2.56248902525490    18.19521175644539    27.72065916946553
H   -1.70640936561209    18.84626520489544    29.13403646686124
H   -2.89593250662947    19.84884175484788    28.30773112995099
C   1.35967257462319     22.61032756843899    27.17500615429325
H   1.87180999656106     22.48443307468614    28.13798946976698
H   2.09824075965422     22.89624693854783    26.42157135028957
H   0.64743597829349     23.43130902728988    27.30604322992857
C   -3.67394195915017    16.26807096735020    30.44073172864062
H   -4.42459029464207    16.72527988255745    29.79048573666226
H   -4.18884783366669    15.69450145999152    31.21926517392438
H   -3.12171054641581    17.06533275745992    30.95398291226432
C   0.42040247878272     13.33639473936136    30.26267772160316
H   0.09653659187276     12.77155771749980    31.14313299004291
H   0.95317365263546     12.66190001989085    29.58613259981379
H   1.12589299744594     14.10122814502953    30.61732081714570
C   -2.07384084077712    17.87634142688515    34.86640390290033
H   -1.79398881699302    18.30991010900273    33.89739108399562
H   -2.86803822648932    17.14971944775522    34.66719449189615
H   -2.46585947233808    18.67330986888787    35.50339283235062
C   1.45263963909707     14.28094184767662    34.79574931629255
H   2.33466747945819     13.94017620326523    35.34293629361253
H   0.74082508568086     13.45292309530115    34.70850552525507
H   1.75722747910068     14.54367564460825    33.77386061056490
[Sn4]2+
168
Sn  7.74828402909865     9.86532591838435     19.05008465848296
N   9.54564899419399     8.79294713853539     21.65784431356814
N   9.88520773072036     7.56813491565985     19.76779245562178
C   9.13014561606400     9.72817782710999     22.65663562182831
C   7.86998656827560     10.21965582716541    24.63844411408278
H   7.18444568254464     9.90956965284881     25.42256349860524
C   9.88329863179046     7.03643375215156     18.43922349687052
C   8.22865055628139     9.30692368716216     23.64454128878481
C   9.66201284066940     11.02551156678004    22.63341449238637
C   8.37963243836805     11.51339956091555    24.63264522534542
H   8.09574778988378     12.20968604702944    25.41636492646875
C   10.53816819503223    7.83921541957974     21.89666425627691
H   11.02893169574681    7.76126329005930     22.85708218358410
C   10.74215652423063    7.10965349492739     20.76937406428848
H   11.44303989625579    6.30528409717803     20.59438354291169
C   9.28000238747389     5.79149385788387     18.20418876858076
C   9.25804565149141     11.91471002382054    23.63267649146037
H   9.66855259616878     12.92137716705223    23.64086552365602
C   9.29879198321258     5.28894361746804     16.90223713455112
H   8.84335915854117     4.32287761500526     16.70105408214516
C   10.51047611773850    7.76481646100549     17.41494408384808
C   9.90841321597270     5.99568969343715     15.87375228196118
H   9.93325406876412     5.57807177418533     14.87137713058922
B   9.10877652139247     8.63724607229156     20.30865323961036
C   10.50591722278140    7.22349177989006     16.12797087843602
H   11.00631289087711    7.75975400767321     15.32587881432351
Sn  5.36992210655244     12.82110068857150    18.02930624297293
N   3.31693930623779     13.61479268347226    20.26035681063999
N   2.89214375575720     15.13063858660994    18.62440724262809
C   3.80861525200353     12.45130778479466    20.91923092625142
C   5.51548864874858     11.33450142207105    22.19835478611982
H   6.42151714638127     11.37635428908014    22.79561124768853
C   2.85549824405663     15.92228889241714    17.43190561210411
C   4.98043673928134     12.52504907666766    21.68467359594132
C   3.15255471244647     11.22816682244892    20.68205517215460
C   4.89761974547203     10.11831476122369    21.96559982927600
H   5.31758194305576     9.20437873082509     22.37637036718003
C   2.26323007701490     14.43083554113099    20.67309735030558
H   1.78028278760579     14.31446382447172    21.63333523657816
C   2.01739720653662     15.34116039993244    19.69428379552733
H   1.28213450744875     16.13388784806691    19.67330715651901
C   3.50033042150522     17.16596478192791    17.42223228738109
C   3.72557517443037     10.05628493961963    21.20361592382544
H   3.20121959158661     9.11231029713627     21.08872131996020
C   3.47941764609179     17.90325381752533    16.23612068412247
H   3.97035169177783     18.87223151466944    16.20501323307424
C   2.18714274857850     15.41632211568839    16.30801392029664
C   2.83310719403475     17.41725255572573    15.10608126034115
H   2.82043302753877     18.00678556016415    14.19448597932763
B   3.73569143637442     14.03191863045205    18.95757914352073
C   2.18971111902954     16.18567827206852    15.14322042084139
H   1.67072475535596     15.81892081456330    14.26185967239746
Sn  5.01329319511895     10.03336314137335    18.52633838470031
N   3.92752843709118     8.03929745709130     16.08899531704803
N   3.27008509255249     7.25734849610013     18.12552210567950
C   4.47604984883348     8.68535118030861     14.93381496243752
C   6.16724697358641     8.77249878776512     13.23546476923310
H   7.12030908704844     8.43759189228785     12.83447874115577
C   2.92587192481290     7.06219511631058     19.49885401169438
C   5.72753486796814     8.26541900769655     14.46055828953440
C   3.68758729007006     9.61368134992876     14.23954329198889
C   5.38604389293700     9.66699987689883     12.51243912345113
H   5.72960726426271     10.03082686673325    11.54853925958734
C   3.23419323683526     6.83575267223885     15.92458920429366
H   3.09026091678369     6.39985895116219     14.94531831535278
C   2.83570206272612     6.37607109910636     17.13504066264868
H   2.28244481999802     5.47924383833638     17.37658965079378
C   3.90995967414114     6.63721185264013     20.40302817603444
C   4.16538149928682     10.09767485454607    13.01976862023698
H   3.55772299549530     10.79566436592200    12.44970980670676
C   3.54650440024711     6.47964672690768     21.74318908801650
H   4.29137494694761     6.13650917099970     22.45691287667304
C   1.59828398799318     7.29165510368212     19.89829512528269
C   2.24706928927606     6.72820329507053     22.16476111201561
H   1.97989287491024     6.59554321202200     23.20866391264424
B   3.97277955983759     8.33208132278344     17.48822572333065
C   1.28125357211792     7.12630905979358     21.24750532865286
H   0.26139090268286     7.30375987681882     21.57779487282238
Sn  8.04340267318939     12.56450318388670    18.82326106257085
N   9.14781968000715     14.19390681879896    16.33271832255664
N   10.79575474913895    14.33566275061857    17.88909428098394
C   7.90490548657064     13.85256292135560    15.72733426592132
C   5.52760783929078     14.20193945346899    15.62104995000992
H   4.66081730794367     14.84701989666606    15.75017806808226
C   11.62260898315996    14.27597026782208    19.05882743194384
C   6.80431670944694     14.68592593911160    15.96151678230812
C   7.78103893141757     12.61086037733907    15.07906421913356
C   5.38487210917444     12.94509969501113    15.00880137130563
H   4.39752926314034     12.58908062943979    14.72510228034342
C   10.20272659248453    14.92647091836846    15.79444822216229
H   10.18209609672508    15.32693806480021    14.79036303593882
C   11.18628289365120    15.01060201046773    16.73228326118084
H   12.14369645571338    15.50921058094165    16.66611592992700
C   11.35822807623668    15.16097691920824    20.11345640587078
C   6.50418870368256     12.17241843917028    14.72569646225848
H   6.38383207190011     11.20987812294296    14.23986310060300
C   12.18632010889801    15.10561977535171    21.23545573143554
H   12.01333476015229    15.79436028004536    22.05794180883798
C   12.67421554198582    13.35121620434406    19.10162041638930
C   13.23921248344987    14.20016279768054    21.29867334493757
H   13.88147656076679    14.17777638037650    22.17392681103341
B   9.49775885151931     13.79423411291420    17.66346508081250
C   13.47725907494729    13.32927670613067    20.24335842696475
H   14.30353800701986    12.62552896332948    20.29670894101628
C   10.69146321406593    11.43495183726545    21.61767805330615
H   10.84716358398905    12.51681297260290    21.62769713622220
H   11.65758046801573    10.96266676897005    21.83407418839176
H   10.42224258068238    11.12866025183620    20.60054777868014
C   7.67964967898097     7.90720076213961     23.64241022923127
H   7.33681201438222     7.61425433660929     22.64295181078736
H   8.44347249291023     7.17765252612868     23.93484022706473
H   6.84782536261035     7.81537201528116     24.34514485524693
C   8.66497761060982     4.99585847112921     19.32069622808502
H   7.90024663191475     4.31607740454356     18.93654632921395
H   9.42147181483095     4.38250147351520     19.82509662806940
H   8.21844738673485     5.64100353032802     20.08202997007526
C   11.21408472063858    9.06858092996337     17.68398379425516
H   10.51357922121668    9.91620437905900     17.70678532441505
H   11.73466791160582    9.06103413104960     18.64625277465036
H   11.94325797200835    9.27606603385093     16.89659504003736
```

```
C   12.94746992145811    12.42312726286663    17.95114870954379
H   13.56252286833908    11.58001254798264    18.27473959308945
H   12.02073845623453    12.03509144856866    17.51631950954756
H   13.48758820529526    12.93560631265341    17.14607338879543
C   10.23267907708623    16.15599515083598    20.03480946244409
H   10.24447073962301    16.70199322419666    19.08576389384412
H   9.25377976642946    15.66403261211661    20.10825445705675
H   10.30227110858457    16.88077386693624    20.84880065172039
C   8.98671869822424    11.76434090932527    14.79526827359845
H   9.56005943471462    11.56816768667911    15.70804890325305
H   8.69265823832478    10.80971209970324    14.35354064576136
H   9.66527738975173    12.27414212911769    14.10236642735317
C   6.96510277749353    16.01697789672412    16.63083394886781
H   6.03328090458483    16.58464733848499    16.58652084901499
H   7.24908385900630    15.89557418784634    17.68527733725900
H   7.76083418174920    16.60305483184716    16.16190932292923
C   2.34685970835819    10.03904266089469    14.77045067002499
H   1.89407071086573    10.79258224308737    14.12164330486848
H   1.65672403592172    9.19032855515238    14.83534695241744
H   2.42770705279341    10.45398102980237    15.78301880425306
C   6.55355963178564    7.27243861445411    15.22760520478753
H   6.86063781940763    7.67092357437856    16.20354471747390
H   5.99615597968700    6.35062599316712    15.42589279565032
H   7.45962867920474    7.01265221815647    14.67785916750071
C   0.53492870482459    7.68890492673800    18.91151671745081
H   0.91598568355706    8.36959290540403    18.14329636190416
H   0.14105959666250    6.81156420381318    18.38530145309118
H   -0.30614479404130    8.16502747727595    19.42155309557761
C   5.31896498087826    6.34160412999384    19.96946354331919
H   5.76543689629498    5.59492530991298    20.63114619758743
H   5.36558577461205    5.96945072464225    18.94244521415306
H   5.95536362897529    7.23772320424003    20.01819556578800
C   4.19242263881585    17.69194408146129    18.64836188689673
H   3.46882424011979    17.98965112628289    19.41561445735483
H   4.83864954521805    16.93274500196352    19.10325992057403
H   4.79830529050394    18.56799254633344    18.40631880978011
C   1.49714493521621    14.08044845258077    16.34852767928407
H   0.88478533028902    13.93557558291963    15.45575334426595
H   2.22178147860936    13.25578773042938    16.39703765248742
H   0.85321455717426    13.98603068970627    17.22882432791049
C   5.63185130647132    13.84104074878198    21.99906870713857
H   6.64216849263971    13.68579570947967    22.38579626198717
H   5.67604654877675    14.49953691412239    21.12677017843058
H   5.05637369522630    14.37351554717333    22.76620321825081
C   1.86558558146807    11.17561755765950    19.91249956890746
H   1.93665273410486    11.71791820972041    18.96187955267636
H   1.57682604335462    10.14111153451808    19.71627123786385
H   1.05760658646968    11.65112376493699    20.48003615671132
Sn4H2
266
Sn  9.61711105650779    3.31873855619906    4.77301207473996
Sn  10.42421405323319    3.20140334025886    7.34988447895956
Sn  10.65648763892582    6.04805453099144    7.35640354786492
Sn  11.08549402587066    5.79709300460208    4.57045250822868
N   8.38803342943865    2.40199979084685    1.86469028594713
N   9.77769080282558    0.69509571623467    2.50594628409846
N   10.75204656951953    1.79011252555979    10.32290664763334
N   8.58306870436952    1.49671252138564    9.65836682757441
N   7.87983873023725    7.96391427552024    7.44885254972360
N   8.77764558935059    7.78305166267253    9.55042803366114
N   13.62005173292530    5.93770338821391    2.38151817260221
N   14.48115293845266    5.89926526224583    4.50129778111222
C   8.48019351808215    1.45776655994366    0.83524300626444
H   7.92769586501498    1.57304782698284    -0.08647661535515
C   9.31163608897942    0.46047649172652    1.20787203380100
H   9.61457188487410    -0.41620469177380    0.65205401595055
C   7.26203567119447    3.29756791874774    1.84490184514945
C   7.38229846664717    4.57829391329509    1.28165312361248
C   6.23037332171365    5.35997346584522    1.17046201158081
H   6.30142591808557    6.35272637404031    0.73683717367532
C   4.99548109367293    4.88550620295943    1.58994950519589
H   4.10946352503142    5.50592241669389    1.48367264345395
C   4.89528458089450    3.62000086460526    2.14769859891070
H   3.92704898028910    3.25546435860796    2.47993257733009
C   6.01948301337548    2.80534160314793    2.28834644881372
C   8.69572273528384    5.07546369375370    0.71847953719920
H   9.50470509853954    4.57981581413605    1.26944380151377
C   8.88584744203673    6.58405633524204    0.87708948234102
H   8.24295425767592    7.15025881064498    0.19210276029416
H   9.92030312722282    6.84960310275779    0.64602419387353
H   8.67405891481125    6.90385433104971    1.90051091949672
C   8.80411489747454    4.68041621148024    -0.76291754353185
H   8.80258725150887    3.59476861088110    -0.89450828602981
H   9.73084833313869    5.07608104675634    -1.18869462139326
H   7.95868634554599    5.09651938649660    -1.32378113364022
C   5.85497296669239    1.41483205659329    2.86898453496461
H   6.84855747381809    0.96412830101132    2.95037349800257
C   5.26353805367954    1.45050478352685    4.28148226003949
H   5.91314101924421    2.00073899582015    4.96957620489051
H   5.15471405920076    0.43230826044390    4.66762964521209
H   4.27431157035205    1.92216110251946    4.29756318288029
C   5.01582782061177    0.52969875751580    1.93699719286606
H   3.99154753791114    0.90893240923717    1.84685496477223
H   4.96210212070167    -0.49225752585421    2.32878136971643
H   5.45140297687155    0.48998942052752    0.93335279675926
C   10.39220310195550    -0.37849326005155    3.23099000782727
C   11.74900994991305    -0.30566320959965    3.58512605262120
C   12.30109635627793    -1.35816746501740    4.31850714611547
H   13.34596019514383    -1.31164016224280    4.61341533306902
C   11.54904898310686    -2.47428604977350    4.64942109332697
H   11.99828505952006    -3.28797247682596    5.21236110681024
C   10.22720642634931    -2.55901050068379    4.23811531599881
H   9.64762428390317    -3.44504747861033    4.48418940492241
C   9.62012847450762    -1.52215272816822    3.52950313163290
C   12.65110640771500    0.80676661010153    3.09411938891935
H   12.02674424657816    1.56572171418032    2.61205302959195
C   13.42089508580069    1.48891667753933    4.22170160923725
H   12.73730194419796    1.88264996790605    4.98064853756350
H   14.00778140824757    2.32380437327105    3.82128663705441
H   14.11717995310433    0.79866341010401    4.71146553505724
C   13.62336861096568    0.24823907926092    2.04334281703759
H   14.27764400926498    -0.51150425291188    2.48542307426480
H   14.25776723610301    1.04933885606287    1.64857701097687
H   13.08722313373868    -0.21732035338991    1.20936900671145
C   8.17767231443338    -1.69893836022835    3.07840770501448
H   7.87341715437095    -0.80473606294781    2.52985857318151
C   8.05684469388052    -2.88371619421595    2.10802430493819
H   8.27845205916101    -3.83322276459872    2.60784053402457
H   8.75147318670287    -2.78085365036855    1.26819595377445
H   7.03862776527631    -2.94323943867405    1.70752093901331
C   7.20489044700365    -1.86246121354937    4.25141678457619
H   6.19070354745016    -2.04258005314288    3.87636632573858
H   7.17768089868250    -0.96191639883673    4.87202194314532
H   7.47806280875534    -2.71004255593954    4.89037561535747
C   10.03546494005714    1.14026565644846    11.33496695805139
H   10.51096615467672    0.84454916182344    12.25986270722689
C   8.75119792308467    0.97221800897615    10.94463407094761
H   7.92932339667413    0.51424163969276    11.47802189424559
C   12.18527662529953    1.80894662004782    10.41840923438198
C   12.83692517511847    2.90785018863661    11.00134044454354
C   14.21391343382257    2.82338424645630    11.21759953940649
H   14.73120166854518    3.65958779133801    11.67851884654138
C   14.92655899619273    1.68662245728628    10.86722654841070
H   15.99565184358205    1.63409994101777    11.05495109285668
C   14.27404505663694    0.62429518409269    10.26057125246159
H   14.84068750751809    -0.25638542840786    9.97018208064307
C   12.90003734718358    0.66370436245883    10.01904975050758
C   12.08136801228992    4.13812745125189    11.45287141447120
H   11.12902138984108    4.16278633097485    10.90910596627326
C   12.82483657556135    5.43954255840803    11.14034052857119
H   12.16339782085192    6.29191944599523    11.31261925799008
H   13.15415776441776    5.47356817764978    10.09770052826346
H   13.70069468304750    5.57199087891149    11.78661969820743
C   11.78076852987447    4.03626340299717    12.95574693350639
H   12.71502573328964    3.92980840800217    13.51970995868554
H   11.14567683236282    3.17278353358462    13.17892232438941
H   11.26979024335195    4.93991833167052    13.30126739445135
C   12.23353327445610    -0.52784136170396    9.35873623934640
H   11.17509743955066    -0.28954411073339    9.21868875456326
C   12.83339711494843    -0.80165147580556    7.97335173603120
H   13.91692222686108    -0.95543703078791    8.02714522370987
H   12.63795822273114    0.03112937831417    7.28952929727471
H   12.39085952185273    -1.70121029812531    7.53340230841164
C   12.31062269573055    -1.77470189397320    10.25038617609025
H   11.75034373974451    -2.59991520026678    9.79572546732050
H   11.88630499857924    -1.57668961715309    11.23988963145325
H   13.34669356166976    -2.10565566319854    10.38597444255923
C   7.37698974777551    1.21056635810154    8.93789745897356
C   6.32802090973850    2.13857851424762    8.91983339845364
C   5.16005635474992    1.80145503921723    8.22911697566802
H   4.33620667413791    2.50927105213220    8.19550584167846
C   5.02633573065255    0.57071000662619    7.60971780189382
H   4.10625021893255    0.32257295956267    7.08812239569185
C   6.06483276813180    -0.35103770886859    7.66330536678887
H   5.94163757646662    -1.31958196743804    7.18850421986658
C   7.25416286086280    -0.05412611767258    8.32723508062251
C   6.40444075073964    3.46308993061923    9.64845266100301
H   7.37310231516958    3.51423119534317    10.15401092958629
C   6.32325159509278    4.63089056475643    8.66524604508840
H   5.36961787328784    4.61924970048670    8.12607851865508
H   7.13083731137273    4.57677439310391    7.92772561192037
H   6.39839047259039    5.59334002859308    9.18559518795129
C   5.30011164776650    3.56580274446400    10.70905178795105
H   5.37173012911854    4.52075369501141    11.23990396843577
H   5.37087459799031    2.75429622592265    11.44145572082605
H   4.30568514365958    3.51854367869350    10.25210636790960
C   8.39119462980348    -1.05865574075997    8.37072383480216
H   8.95610124060578    -0.88243932290238    9.29384680580317
C   9.34138343335991    -0.84441784297507    7.18576840399096
H   9.77213356378603    0.16165962697859    7.18460778474327
H   10.16079754652317    -1.56899474990961    7.21185350830336
H   8.81558114429564    -0.97947456106093    6.23875850614118
C   7.91513716193828    -2.51424448638685    8.40795477232317
H   7.18199267984687    -2.68037488263877    9.20428958595651
H   7.46378053491147    -2.82131743468953    7.45816412475280
H   8.77087454849055    -3.17425707205788    8.58567291769116
C   7.07514908472891    8.65373925155386    8.36120786845531
H   6.19014813681121    9.18619628945086    8.04068052649032
C   7.60309389764211    8.54787899463764    9.59996690675311
H   7.24991018493561    8.97570624803442    10.52735444016285
C   7.71678997923026    8.25067333055809    6.04984379265825
C   6.80647277116197    7.52076820430692    5.27079238197277
C   6.69778638156979    7.83102772495062    3.91379648308963
H   6.01978722757069    7.25394964324985    3.29124831185805
C   7.43457159000994    8.86329307358522    3.35357906998882
H   7.34270798119209    9.08627789691026    2.29407711107926
C   8.27145045694051    9.62615948309115    4.15435807433682
H   8.81793508781467    10.45847833469399    3.71901889068676
C   8.41189796372769    9.34995100373680    5.51434960861586
C   5.89131744694343    6.47661901961230    5.86946463824682
H   6.12598293094218    6.40556417371289    6.93315396641953
C   6.10045783330483    5.10211762964684    5.23351448411677
H   5.42030828477418    4.36182124266280    5.67026675243096
H   7.12600949650790    4.75080716982638    5.39378895551328
H   5.91707504298669    5.12996636565428    4.15558536135319
C   4.42304243475486    6.91179303970631    5.74451712646143
H   4.10993827475964    6.94546178634523    4.69471107248861
H   4.26103336079303    7.90559664643862    6.17586221376311
H   3.77255298187148    6.19963802170824    6.26534528636085
C   9.26832762516382    10.25985680233090    6.37163563746934
H   9.13209085026497    9.97122844104346    7.41835485380720
C   10.74755832163082    10.09761488891895    6.02335478186500
H   11.06925703848778    9.06088848368154    6.17733616973885
H   11.37130302643379    10.75075974582331    6.64364680339242
H   10.93188730208782    10.35006970033475    4.97380214805043
C   8.83038191049995    11.72556219394120    6.24895899957512
H   9.01123284297478    12.11938020594069    5.24300299066265
H   9.39306582249866    12.34596994989440    6.95521036863094
H   7.76368666758301    11.83694331267512    6.46888085969572
C   9.60656085640260    7.78317777760765    10.72400975487034
C   9.19549936414065    7.07618926403229    11.87044028982013
C   9.91541178343136    7.25217733675612    13.05494348122334
H   9.59437243883263    6.73065531683992    13.95228038013600
C   11.01766263249160    8.08827281442426    13.11268855492467
H   11.56117965200244    8.21400518122772    14.04491018378102
C   11.42633659001483    8.75913665965854    11.97032048944167
H   12.29282135415738    9.41155864194679    12.01798253818015
C   10.73613894780289    8.62851810497646    10.76476193858471
C   7.97903617600110    6.16807532686281    11.89726563690735
H   7.60592835554958    6.07211803949068    10.87272443177469
C   8.33426096591965    4.76484661115851    12.40782281465334
H   8.71515691666591    4.79592468672108    13.43374391430786
H   9.09666204861881    4.29049299552729    11.78269961396238
H   7.44849778135024    4.12251522495578    12.41061073726706
C   6.86755681314012    6.77934928173652    12.76507748866321
H   5.97798090201513    6.14030046753538    12.76207338606410
H   6.57423631274673    7.77141496271556    12.40931373563712
H   7.19956974286200    6.88280070609249    13.80430524291084
```

```
  C   11.17219502796023     9.47188301389336     9.58298472444031
  H   10.74854746560122     9.03099791024871     8.67567352157551
  C   12.69235275720911     9.49458847333867     9.39813736703337
  H   13.19498262343255    10.06876110583410    10.18480349206485
  H   12.94215011478824     9.96419320929962     8.44088025403842
  H   13.10277017184551     8.48041173871292     9.38721670844630
  C   10.61243139395606    10.89480070492606     9.72901180774816
  H   9.51843367812907    10.88820974857463     9.78293423936030
  H   10.91165406861051    11.51698980827304     8.87788160227058
  H   10.99611402647851    11.36268845526993    10.64328077761541
  C   15.02009242911771     5.94883707195490     2.32004093591289
  H   15.54054729929846     5.98452295821984     1.37332846774569
  C   15.52565348442144     5.92861269787757     3.57133887558296
  H   16.55947629352275     5.94837744216514     3.88659803942812
  C   12.92464318486159     6.25022623386270     1.16741100993583
  C   12.58774144053097     7.59512800903554     0.90908840771107
  C   12.11287243950134     7.92518114983481    -0.36058365039098
  H   11.86115099089527     8.95754162135154    -0.58422312879913
  C   11.97448659702610     6.96191776910174    -1.34887927334430
  H   11.61754472321984     7.24125576251029    -2.33633894152422
  C   12.28360385018333     5.64054526751933    -1.06897521480832
  H   12.17111244031275     4.89111499346527    -1.84745774266360
  C   12.75567090606166     5.25612882540819     0.18861841760811
  C   12.80445131612677     8.69884359443848     1.92712294183599
  H   12.90947776130145     8.23461118488945     2.91417589278447
  C   11.61898575453203     9.66552013120375     2.01150505842082
  H   11.49095021811821    10.24271892579535     1.08921892943743
  H   11.78964583819981    10.38389291748711     2.82075417911132
  H   10.68752147515879     9.12861816318771     2.21959668346090
  C   14.10649243541967     9.45317266354771     1.61601607779140
  H   14.96687006616325     8.77621755493808     1.62514243807688
  H   14.28211707091583    10.24020539481392     2.35804420050111
  H   14.05131224206703     9.92364702269885     0.62722572242452
  C   13.12074938095597     3.80138380180866     0.42309042776697
  H   13.36877314341002     3.67942752667959     1.48456379244167
  C   11.94630781095145     2.86874544189932     0.09912724612841
  H   11.63296234821651     2.96123662668249    -0.94597936178070
  H   11.07791234097379     3.08051326978368     0.73045783269829
  H   12.23297586186972     1.82568490394937     0.26099818760569
  C   14.35500889589693     3.41825921403116    -0.40801872953983
  H   14.63401720060233     2.37453002416863    -0.22799647041981
  H   15.21523966698161     4.04862651243871    -0.16281135026026
  H   14.15151924522071     3.52978105776622    -1.47904627779122
  C   14.79005907311536     6.21288476365950     5.86930300167545
  C   15.09253605323454     7.54591653410451     6.19864623062375
  C   15.41746495455114     7.83954529911423     7.52475379244377
  H   15.64413250436839     8.86512828355117     7.80387524772813
  C   15.45538584830269     6.84483276686617     8.48930297406280
  H   15.69440048868163     7.09352812779041     9.51997144423406
  C   15.19864957966859     5.52685701590853     8.13605397226748
  H   15.25080026261316     4.75101157882088     8.89345657671315
  C   14.87572789495090     5.18586740204339     6.82293469307958
  C   15.13263582573452     8.65359775730907     5.16137704424481
  H   14.76758375741773     8.24721944271053     4.21366545212730
  C   14.22661892984967     9.82651364225852     5.54191278171528
  H   13.20610674712163     9.48069662261128     5.71362880404074
  H   14.20891039008977    10.57407705612331     4.74070785181457
  H   14.57431110780855    10.32750569212942     6.45221035339912
  C   16.57300192381793     9.13424511848665     4.93172014026254
  H   16.98644044556236     9.58360359913517     5.84189856196223
  H   16.59851476948521     9.89004228438080     4.13843731790364
  H   17.22854716685186     8.30855675854168     4.63812356981631
  C   14.71346124624630     3.74012993087422     6.41123028786679
  H   13.92836538273222     3.70544831522693     5.64863906812125
  C   14.30698977125929     2.82213268167971     7.55641402137140
  H   15.12060093188084     2.67702535825586     8.27366638324778
  H   14.04519742562734     1.83564424675331     7.16453156081780
  H   13.44098851844755     3.21018838926640     8.10320118192771
  C   16.02537802500760     3.22907595366069     5.78805946924137
  H   16.26650494870672     3.74802511764011     4.85628748467819
  H   15.95488375857761     2.15837742368748     5.56991866371648
  H   16.85341226174666     3.37860318645684     6.49100683997050
  B   9.23702680014310     1.96608128624577     2.96391881744997
  B   9.84555799884921     2.05162503193187     9.21926908188949
  B   8.99513023773249     7.37476067873799     8.16856515518282
  B   13.21881206713159     5.89828460768249     3.77782566990385
  H   10.15030357990409     6.94382364037474     3.67311769182387
  H   12.13442701528172     6.48606234624703     8.14245510450601
C6H6
12
  C   0.27135062800167    -0.00000000130327    -1.93444040638351
  C   -0.88661534434177    -0.35534068272227    -1.23507965139211
  C   1.42931659720316     0.35534068447640    -1.23507965308489
  H   -1.79259123859904    -0.63319413954182    -1.78217723126627
  H   2.33529249124253     0.63319414143275    -1.78217723459668
  C   -0.88661534623033    -0.35534068088393     0.16359256256190
  C   1.42931659868691     0.35534068558786     0.16359256213566
  H   -1.79259123712410    -0.63319414207152     0.71069014998241
  H   2.33529249410008     0.63319414226017     0.71069014720328
  C   0.27135062576977     0.00000000044732     0.86295330759449
  H   0.27135062422471    -0.00000000524689     1.95716914289173
  H   0.27135063106641    -0.00000000243478    -3.02865624764601
C4H4
8
  C   -1.00122487329754    -0.01014963349991    -1.34457232140451
  C   0.09812762346456     0.11630915701889    -0.22318977508835
  C   -0.84286911502898     0.00806571137301     0.71154133353634
  C   -1.94214059772948    -0.11909220645109    -0.40984116129062
  H   -3.01681832318474    -0.24254942045485    -0.41699203372358
  H   -0.99414154703171    -0.00933562468115    -2.42632314995763
  H   1.17283509682043     0.23949683545467    -0.21600669145090
  H   -0.85001620001254     0.00752074724043     1.79329288337924
[C4H4]2+
8
  C   -0.91543894211707     0.00095112847697    -1.34066028728928
  C   0.09557008201526     0.11456534461425    -0.30974886923982
  C   -0.92891615127391    -0.00056561800517     0.70761066687295
  C   -1.93928062751078    -0.11981616049740    -0.32330418200011
  H   -3.04009720057496    -0.24865601293073    -0.33067011629548
  H   -0.90851641958879     0.00395339758915    -2.44899336656341
  H   1.19688905175871     0.23903099903071    -0.30226748253077
  H   -0.93645772870845     0.00080248772222     1.81594272104591
[C4H4]2-
8
  C   -0.91521266199973     0.00020195986459    -1.35720320511753
  C   0.11153489800536     0.12052725552491    -0.30967007553001
  C   -0.92876803241230    -0.00255723239938     0.72408420719897
  C   -1.95553700892709    -0.12261248432700    -0.32344063338679
  H   -3.06003565776150    -0.24647929866115    -0.33083781806443
  H   -0.90819585719344     0.00022760935673    -2.46864021575531
  H   1.21615690596178     0.24329344374332    -0.30190045049538
  H   -0.93619052167308    -0.00233568710202     1.83551727515048
K2Sn4_ph
122
Coordinates from ORCA-job ph
  Sn  7.76763318715938     9.08204990921414    19.52744441171072
  K   4.70035038690948    10.24984890012391    21.15201877396584
  N   11.04843476584286     8.31656590378346    19.51080112620519
  N   10.19232539999809     7.51511224584385    17.55780969298242
  C   11.31080939222485     8.85048195198959    20.79262375790530
  C   12.41222818291841     8.71416306430530    22.93943089109137
  H   13.05743002970491     8.17371343307742    23.62638352041317
  C   9.46560470916305     7.24827730384297    16.38203421762530
  C   12.14173189257328     8.17003154146667    21.68971036334895
  C   10.74741319621801    10.06827311933418    21.17646882579099
  C   11.85060669530271     9.93063983803958    23.31957382837878
  H   12.06392737406521    10.35082427697820    24.29774835693605
  C   12.04187399883084     7.61341924573459    18.82031282176483
  H   13.04830024015271     7.51679938020970    19.20160566225905
  C   11.54410806727050     7.15462690132244    17.65463489964786
  H   12.03759735774260     6.56358314349651    16.89620967472424
  C   8.12921559714819     6.84999223144701    16.45137023587743
  C   11.01099790114824    10.59823049298488    22.43391341393339
  H   10.56166359996845    11.54808649591439    22.71038970727198
  C   7.39423925793473     6.66073211242925    15.28524577654545
  H   6.34775722878810     6.38230736797882    15.36206363792720
  C   10.06294746218653     7.42351674786520    15.12350520121439
  C   7.98461878468196     6.83792988664524    14.03779186172527
  H   7.40652547448052     6.68341112314790    13.13196412500499
  B   9.80053983202084     8.28958676242547    18.74352330251241
  C   9.32550180249482     7.21378770595595    13.96298397235246
  H   9.80023106106228     7.35307390058390    12.99538554117250
  Sn  5.49459813248150    11.89361827315240    17.72501278809719
  N   3.05933549417448    13.72011620814394    19.27901298049698
  N   4.19667883616447    14.97081616816016    17.76329801570449
  C   2.54954705904929    12.74161283979711    20.15336698807097
  C   2.00134525722520    12.02182267497576    22.39970828550776
  H   1.99155352712881    12.21526397174396    23.46906635106266
  C   4.99513582083711    15.51280611174728    16.72311705648017
  C   2.54188629947083    12.97016410859303    21.53508820841427
  C   2.02755012910466    11.54449509481549    19.65185603882099
  C   1.46979570761649    10.83508920377519    21.89633408253556
  H   1.03102541379116    10.10586748585793    22.57098571621920
  C   2.66125412698413    15.06011835741407    19.39752103798368
  H   1.90505294108666    15.37271031046905    20.10373696501636
  C   3.32194133574620    15.79593636744344    18.47915907915034
  H   3.25840479406137    16.85822806200356    18.29038620625197
  C   6.36900445641612    15.27337907900830    16.68134559684471
  C   1.48968331254508    10.59942722227116    20.52154557412764
  H   1.07296722931469     9.68069009037587    20.11644434205450
  C   7.13912834223261    15.83340195714444    15.66767619207327
  H   8.21099833145809    15.65502961967246    15.66456218723338
  C   4.40442667956580    16.31790840303058    15.74625834105352
  C   6.55353825589416    16.63857207385592    14.69541412815609
  H   7.16019231366247    17.08196882645411    13.91123229754252
  B   4.07628163980685    13.60400455065862    18.24272554946054
  C   5.18280178563901    16.87929701539338    14.74030112843206
  H   4.71243207488977    17.50115364655071    13.98387937150217
  Sn  5.37130313943983     9.07981299301507    17.65907737081371
  K   8.44174480216455    10.23727976984089    16.02771172178465
  N   2.08918175251532     8.32204785764132    17.66797739288454
  N   2.93254083848990     7.52977796452476    19.63037775148531
  C   1.83393701794111     8.84957023467892    16.38194512320305
  C   0.74487419100223     8.70143080114971    14.22970992279872
  H   0.10421907856984     8.15692379297043    13.54171109800850
  C   3.65080178899697     7.27360380626826    20.81337029983795
  C   1.00901856997144     8.16366958933542    15.48353255845178
  C   2.39783548998035    10.06628067277416    15.99566081910889
  C   1.30709330519590     9.91683224540154    13.84698502497216
  H   1.09874990004223    10.33203901652801    12.86561488279328
  C   1.08990594526510     7.62650944313744    18.35768089043199
  H   0.08534723493654     7.53030977318222    17.97136318801541
  C   1.58007067197702     7.17319632376912    19.52865640272155
  H   1.08063324676781     6.58760832695123    20.28738924309255
  C   4.99052019567266     6.88396473572039    20.75662064700415
  C   2.14072882524845    10.58984310893606    14.73417402873326
  H   2.59030721644715    11.53900529964915    14.45572057865960
  C   5.71867724286932     6.70839388209268    21.92914519164979
  H   6.76773799980963     6.43740766004442    21.86117242004368
  C   3.04299624735135     7.45180307299848    22.06648509622909
  C   5.11798721498137     6.88976681530028    23.17121593599113
  H   5.69052553903390     6.74577201250617    24.08230081338667
  B   3.33305773283465     8.29587459975312    18.44152015423807
  C   3.77361303954080     7.25524660123655    23.23360925379956
  H   3.29073388927827     7.39727360645037    24.19676278010338
  Sn  7.64809459155739    11.89733127382088    19.44776043932532
  N   10.08583559791892    13.70926266010352    17.87666334515240
  N   8.94965271313102    14.97231756316224    19.38300992919393
  C   10.59484524114452    12.72463136308350    17.00893181803289
  C   11.14336565735666    11.99022981401615    14.76723788860560
  H   11.15390545817015    12.17703704599502    13.69670600296420
  C   8.15127031905411    15.52370338698606    20.41830566404142
  C   10.60344368729749    12.94445872615098    15.62570524931095
  C   11.11523321267549    11.52998787635206    17.51810471456157
  C   11.67329408225143    10.80602534684810    15.27822543015533
  H   12.11186102369565    10.07218674306919    14.60840762885279
  C   10.48493307726731    15.04808280015838    17.74805097284476
  H   11.24132821040411    15.35466483058556    17.03941901191880
  C   9.82510267637493    15.79128806147561    18.66095586934182
  H   9.88940293564700    16.85494300547738    18.84158304487584
  C   6.77715409523778    15.28608352888208    20.46187283823659
  C   11.65239354495422    10.57896496096490    16.65442994279357
  H   12.06736148796696     9.66198134613825    17.06516549421480
  C   6.00735695866531    15.85597672829071    21.47027132005876
  H   4.93528596340815    15.67888536768142    21.47461470640113
  C   8.74249347202458    16.33678935069154    21.38824724599650
  C   6.59346412039105    16.66917702094900    22.43549061900961
  H   5.98698470885202    17.12021605150878    23.21543224994911
  B   9.06919305438124    13.60154255449702    18.91444363069687
  C   7.96449173386623    16.90796270402299    22.38893504670624
  H   8.43533965313348    17.53602561999633    23.13990414235566
  H   2.96941349318506    13.89028957560881    21.92341298959884
  H   2.02041641243789    11.37282277938675    18.58091261376865
  H   2.00472338285650     7.76495571733430    22.11763966119151
  H   5.45935243672360     6.73533884646741    19.79003208206553
  H   3.03253599430441    10.60642406128891    16.69171576528705
  H   0.59777033586672     7.19656255897310    15.75682615357142
  H   7.66871525949308     6.70466156282068    17.42249967811824
```

```
  H   11.09866448342171      7.74377879630779     15.06311046857258
  H   12.55252214854557      7.20201969230819     21.41901238117799
  H   10.10794410888302     10.60434614962205     20.48158480766223
  H   6.30835517377393     14.66917416104493     19.70294487244768
  H   9.81608145750608     16.50241191215437     21.36275917297063
  H   10.17724365301343     13.86264234735644     15.23135992847590
  H   11.12157083606734     11.36491623042737     18.59009555625738
  H   3.33102520941326     16.48488751396708     15.77058368601362
  H   6.83723347101137     14.66277423050660     17.44570295538012
K2Sn4_me
66
  Sn  7.92576951037594      9.73193007035270     18.18509546218917
  K   6.55414317421685     11.10635269650887     21.82939876545354
  N   9.63680904907038      8.18346517589968     20.72835420073343
  N   10.09297538278530      7.30447878236934     18.68528442600428
  C   10.56104919925015      7.13861150850051     20.87206022418845
  H   10.95088463163717      6.85103769426219     21.84823551738267
  C   10.83687438977525      6.61678127502408     19.64505713111448
  H   11.50518908362881      5.79794732343314     19.37948769194074
  B   9.29511753398487      8.33549735261520     19.32259765398947
  Sn  5.20402274799378     12.46053568242352     18.14995331440034
  N   3.47952599765263     14.02224654030119     20.68210357431224
  N   3.04286740224527     14.89328822440037     18.63165952587223
  C   2.55527046894503     15.06878762860952     20.81314972995277
  H   2.15728355103956     15.36094244034422     21.78466580155503
  C   2.29112748271142     15.58586453073989     19.58158754000070
  H   1.62603227254166     16.40427401423699     19.30677295084917
  B   3.83400903248057     13.86376099015554     19.28011429967907
  Sn  5.19568839494348      9.73871701187070     19.00454492412866
  K   6.58091880473471     11.08186845546114     15.34041718637115
  N   3.49449867177458      8.17545906910876     16.45785967403234
  N   3.03995422509247      7.30525689370372     18.50490728814939
  C   2.57234059201762      7.12803986492057     16.31904926753963
  H   2.18264031309090      6.83555685013973     15.34428572975362
  C   2.29781428357513      6.61124506534062     17.54850165547495
  H   1.63046678689555      5.79287168944476     17.81793170763874
  B   3.83619984308219      8.33486491997892     17.86301995520827
  Sn  7.92527441137656     12.46096374376600     19.00002724104799
  N   9.66455571185043     13.99952681834693     16.46901576063842
  N   10.10573057041059     14.87883178935120     18.51516712874279
  C   10.59416596296520     15.04055967165296     16.33258627435044
  H   10.99396792772285     15.32544701138361     15.35966253462447
  C   10.86058552423572     15.56294993120052     17.56144614579150
  H   11.52965006519386     16.37949626158368     17.83213661701537
  B   9.30961212403116     13.85022884643794     17.87174923758003
  C   10.19943791345520     15.22668885110437     19.91920911338155
  H   9.52247248744702     14.58147424801741     20.48799215561973
  H   11.21983938238512     15.08098559605557     20.29946367461287
  H   9.90915515390779     16.27189207411946     20.09142098421861
  C   9.23327987908693     13.22670642571419     15.32822118372942
  H   9.94901861399406     13.32685405986103     14.50419205613773
  H   9.19021722811374     12.16516261667106     15.60915419327251
  H   8.24646843912636     13.55701557842630     14.95862563449128
  C   2.94985827416762     15.23427332756771     17.22582993319108
  H   3.62452677642390     14.58407537001483     16.66001815714409
  H   1.92886146751277     15.08996026441281     16.84660502373236
  H   3.24338872801743     16.27768727335900     17.04834026944463
  C   3.90561519943857     13.25210035124509     21.82663720159715
  H   3.18398766302835     13.35026030936275     22.64581806373491
  H   3.95358330175962     12.19066656368876     21.54632282284868
  H   4.88890202233647     13.58572085811279     22.20296260791071
  C   3.90437456845899      8.96999536442489     15.32386044013711
  H   4.86611646762877      8.62513235942087     14.90354428645210
  H   3.15309427917430      8.91945195610885     14.52717049210787
  H   3.98948930102803     10.01927314332363     15.63802836884184
  C   2.94420262869861      6.95614923805293     19.90864524502057
  H   1.91650744912124      7.07229955700231     20.27902544971935
  H   3.26371147409595      5.92017221150335     20.08520447941703
  H   3.59700435310968      7.62148499704225     20.48247732206458
  C   10.18962284438340      6.95023460250098     17.28296133535744
  H   11.21634016337159      7.07043294717300     16.91114606930334
  H   9.87552655337521      5.91187281552057     17.11083664141792
  H   9.53275580333580      7.60978375285647     16.70707928962628
  C   9.22348220709568      8.98094459801056     21.85935822556824
  H   8.25459700264123      8.64438217470254     22.26950458551686
  H   9.96656127348923      8.92180554086358     22.66305032969004
  H   9.15082062693273     10.03160948243466     21.54647686827190
```

3. References for supporting information

s1 (a) F. Neese, *WIREs Computational Molecular Science* **2012**, *2*, 73-78; (b) F. Neese, F. Wennmohs, U. Becker, C. Riplinger, *J. Chem. Phys.* **2020**, *152*, 224108; (c) F. Neese, *WIREs Computational Molecular Science* **2018**, *8*, e1327.

s2 (a) J. W. Furness, A. D. Kaplan, J. Ning, J. P. Perdew, J. Sun, *J. Phys. Chem. Lett.* **2020**, *11*, 8208-8215; (b) J. W. Furness, A. D. Kaplan, J. Ning, J. P. Perdew, J. Sun, *J. Phys. Chem. Lett.* **2020**, *11*, 9248-9248.

s3 S. Grimme, A. Hansen, S. Ehlert, J.-M. Mewes, *J. Chem. Phys.* **2021**, *154*, 064103.

s4 (a) E. Caldeweyher, C. Bannwarth, S. Grimme, *J. Chem. Phys.* **2017**, *147*, 034112; (b) E. Caldeweyher, S. Ehlert, A. Hansen, H. Neugebauer, S. Spicher, C. Bannwarth, S. Grimme, *J. Chem. Phys.* **2019**, *150*, 154122.

s5 H. Kruse, S. Grimme, *J. Chem. Phys.* **2012**, *136*, 154101.

s6 P. V. R. Schleyer, C. Maerker, A. Dransfeld, H. Jiao, N. J. R. Van Eikema Hommes, *J. Am. Chem. Soc.* **1996**, *118*, 6317-6318.

s7 (a) F. Weigend, *Phys. Chem. Chem. Phys.* **2006**, *8*, 1057; (b) F. Weigend, R. Ahlrichs, *Phys. Chem. Chem. Phys.* **2005**, *7*, 3297; (c) S. Ehlert, U. Huniar, J. Ning, J. W. Furness, J. Sun, A. D. Kaplan, J. P. Perdew, J. G. Brandenburg, *J. Chem. Phys.* **2021**, *154*, 061101.

s8 S. T. Howard, T. M. Krygowski, *Can. J. Chem.* **1997**, *75*, 1174-1181.

s9 (a) M. Giambiagi, M. S. De Giambiagi, K. C. Mundim, *Struct. Chem.* **1990**, *1*, 423-427; (b) M. Giambiagi, M. Segre De Giambiagi, C. D. Dos Santos Silva, A. Paiva De Figueiredo, *Phys. Chem. Chem. Phys.* **2000**, *2*, 3381-3392.

s10 T. Lu, F. Chen, *J. Comp. Chem.* **2012**, *33*, 580-592.

s11 S. Noorizadeh, E. Shakerzadeh, *Phys. Chem. Chem. Phys.* **2010**, *12*, 4742.

s12 E. Matito, *Phys. Chem. Chem. Phys.* **2016**, *18*, 11839-11846.

s13 J. K. B. E. D. Glendening, A. E. Reed, J. E. Carpenter, C. M. M. A. Bohmann, P. Karafiloglou, C. R. Landis, and F. Weinhold, Theoretical Chemistry Institute, University of Wisconsin Madison, **2018**.
